# CRISPR Screening and Comparative LC‐MS Analysis Identify Genes Mediating Efficacy of Delamanid and Pretomanid against *Mycobacterium tuberculosis*

**DOI:** 10.1002/advs.202400176

**Published:** 2024-08-20

**Authors:** Mei‐Yi Yan, Haifeng Li, Yun‐Mo Qu, Si‐Shang Li, Dandan Zheng, Xiao‐Peng Guo, Zhaojun Wu, Jie Lu, Yu Pang, Weimin Li, Jian Yang, Lingjun Zhan, Yi‐Cheng Sun

**Affiliations:** ^1^ NHC Key Laboratory of Systems Biology of Pathogens, State Key Laboratory of Respiratory Health and Multimorbidity National Institute of Pathogen Biology and Center for Tuberculosis Research Chinese Academy of Medical Sciences & Peking Union Medical College Beijing 100730 P. R. China; ^2^ NHC Key Laboratory of Human Disease Comparative Medicine, and National Center of Technology Innovation for Animal Model Institute of Laboratory Animal Sciences Chinese Academy of Medical Sciences & Peking Union Medical College Beijing 100021 P. R. China; ^3^ Beijing Tuberculosis and Thoracic Tumor Research Institute Beijing Chest Hospital Capital Medical University Beijing P. R. China; ^4^ Beijing Key Laboratory for Pediatric Diseases of Otolaryngology, Head and Neck Surgery Beijing Pediatric Research Institute Beijing Children's Hospital Capital Medical University National Center for Children's Health Beijing P. R. China; ^5^ Department of Bacteriology and Immunology Beijing Chest Hospital Capital Medical University Beijing P. R. China

**Keywords:** antibiotic resistance, CRISPR screening, delamanid, *Mycobacterium tuberculosis*, pretomanid

## Abstract

Tuberculosis (TB), the leading cause of death from bacterial infections worldwide, results from infection with *Mycobacterium tuberculosis* (Mtb). The antitubercular agents delamanid (DLM) and pretomanid (PMD) are nitroimidazole prodrugs that require activation by an enzyme intrinsic to Mtb; however, the mechanism(s) of action and the associated metabolic pathways are largely unclear. Profiling of the chemical‐genetic interactions of PMD and DLM in Mtb using combined CRISPR screening reveals that the mutation of *rv2073c* increases susceptibility of Mtb to these nitroimidazole drugs both in vitro and in infected mice, whereas mutation of *rv0078* increases drug resistance. Further assays show that Rv2073c might confer intrinsic resistance to DLM/PMD by interfering with inhibition of the drug target, decaprenylphophoryl‐2‐keto‐b‐D‐erythro‐pentose reductase (DprE2), by active nicotinamide adenine dinucleotide (NAD) adducts. Characterization of the metabolic pathways of DLM/PMD in Mtb using a combination of chemical genetics and comparative liquid chromatography‐mass spectrometry (LC‐MS) analysis of DLM/PMD metabolites reveals that Rv0077c, which is negatively regulated by Rv0078, mediates drug resistance by metabolizing activated DLM/PMD. These results might guide development of new nitroimidazole prodrugs and new regimens for TB treatment.

## Graphical abstract

This work utilizes combined CRISPR screening to identify genes that affect the efficacy of delamanid (DLM) and pretomanid (PMD) in *Mycobacterium tuberculosis* (Mtb). Along with chemical‐genetic profiling, comparative liquid chromatography‐mass spectrometry analysis characterizes the metabolic pathways of DLM and PMD in Mtb, providing insights for developing new antitubercular drugs and targeted regimens for drug‐resistant tuberculosis.

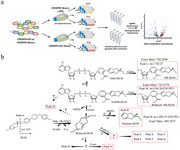

## 1 Introduction

Tuberculosis (TB), caused by infection with the bacillus *Mycobacterium tuberculosis* (Mtb), remains the leading cause of death from bacterial infections. Therapy for drug‐sensitive TB is complex, requiring at least 6 months of treatment with up to four drugs.^[^ ^1^, ^2^ ^]^ Multidrug‐resistant TB (MDR‐TB) and extensively drug‐resistant TB (XDR‐TB) are even more difficult to treat. Various mechanisms are responsible for Mtb resistance to different drugs.^[^ ^3^, ^4^, ^5^, ^6^, ^7^ ^]^ Exploring these drug‐resistance mechanisms might not only help to slow or prevent emergence of drug‐resistant TB, but also facilitate design of new regimens to shorten the duration of TB chemotherapy.

The bicyclic 4‐nitroimidazole drugs delamanid (DLM) and pretomanid (PMD), a novel class of antimicrobial agents used to treat TB, have been approved recently as a treatment for MDR‐TB.^[^ ^8^, ^9^, ^10^, ^11^, ^12^ ^]^ DLM and PMD are prodrugs that require activation by the deazaflavin (F_420_)‐dependent nitroreductase Ddn (Rv3547) in Mtb to exert their bactericidal activities.^[^ ^9^, ^13^ ^]^ It is thought that both drugs act in three ways: they 1) interfere with the synthesis of mycolic acid,^[^ ^8^, ^9^ ^]^ 2) are respiratory poisons,^[^ ^13^, ^14^, ^15^, ^16^ ^]^ and 3) repress arabinogalactan synthesis.^[^ ^17^ ^]^ Decaprenylphophoryl‐2‐keto‐b‐D‐erythro‐pentose reductase (DprE2), an essential enzyme required for synthesis of the cell‐wall component arabinogalactan,^[^ ^18^, ^19^ ^]^ was identified recently as a molecular target of DLM and PMD.^[^ ^17^ ^]^ DLM inhibits synthesis of methoxy mycolic acid and ketomycolic acid, whereas PMD inhibits synthesis of ketomycolic acid, a key component of mycobacterial cell walls.^[^ ^8^, ^9^ ^]^ To date, however, the mechanism by which DLM and PMD block mycolic acid synthesis remains unclear.

Metabolic activation of DLM or PMD by Ddn generates one major metabolite, des‐nitroimidazole (des‐nitro), which has no antimycobacterial activity.^[^ ^13^, ^20^ ^]^ Because NO may be produced during this process, DLM and PMD might function as specific NO donors that react with cytochromes and cytochrome c oxidase to interrupt coupling of respiration to oxygen reduction.^[^ ^13^, ^21^, ^22^ ^]^ Consistent with this hypothesis, transcriptional profiling of Mtb revealed that DLM or PMD affect genes that respond to respiratory poisoning.^[^ ^14^, ^15^, ^23^ ^]^ In addition, formation of adducts of DLM and PMD with nicotinamide adenine dinucleotide (NAD) may inhibit DprE2 activity, thereby providing a mechanism responsible for anti‐mycobacterial activity.^[^ ^17^, ^20^, ^24^ ^]^ It is suggested that more than one mechanism might be involved in growth inhibition and killing of Mtb, with these NAD adducts being more important for killing than for growth inhibition.^[^ ^24^ ^]^

Because PMD and DLM belong to the same class of bicyclic nitroimidazole prodrugs, Mtb is often cross‐resistant to both.^[^ ^12^, ^25^ ^]^ Mutation of genes required for F_420_ cofactor biosynthesis and recycling (e.g., *ddn*, *fgd1*, *fbiA*, *fbiB*, *fbiC*, and *fbiD*) might affect drug activation, leading to resistance of Mtb to DLM and PMD.^[^ ^26^, ^27^, ^28^, ^29^ ^]^ Although less is known about other mechanisms responsible for resistance to DLM and PMD, a recent study showed that *cinA* is an intrinsic PMD resistance gene in Mtb, suggesting that its encoded protein, CinA, might be involved in degradation of NAD adducts.^[^ ^24^ ^]^ Other intrinsic resistance genes may also be involved in degradation of active metabolites in Mtb. Identification of these genes and investigation of their roles might help to determine the mechanism by which DLM and PMD kill Mtb.

Here, we used a recently developed combined CRISPR screening platform to identify dozens of genes that might affect the efficacy of DLM and PMD in Mtb.^[^ ^30^ ^]^ We then combined chemical genetics analyses with liquid chromatography‐mass spectrometry (LC‐MS) analysis of DLM/PMD metabolites from different Mtb mutants to characterize pathways by which Mtb metabolizes DLM/PMD. These findings might aid development and optimization of new antitubercular drugs, as well as that of direct treatment regimens for drug‐resistant TB.

## 2 Results

### 2.1 Combined CRISPR Screening Identifies Genes That Determine the Antimicrobial Efficacy of DLM and PMD against Mtb

**Figure 1:**
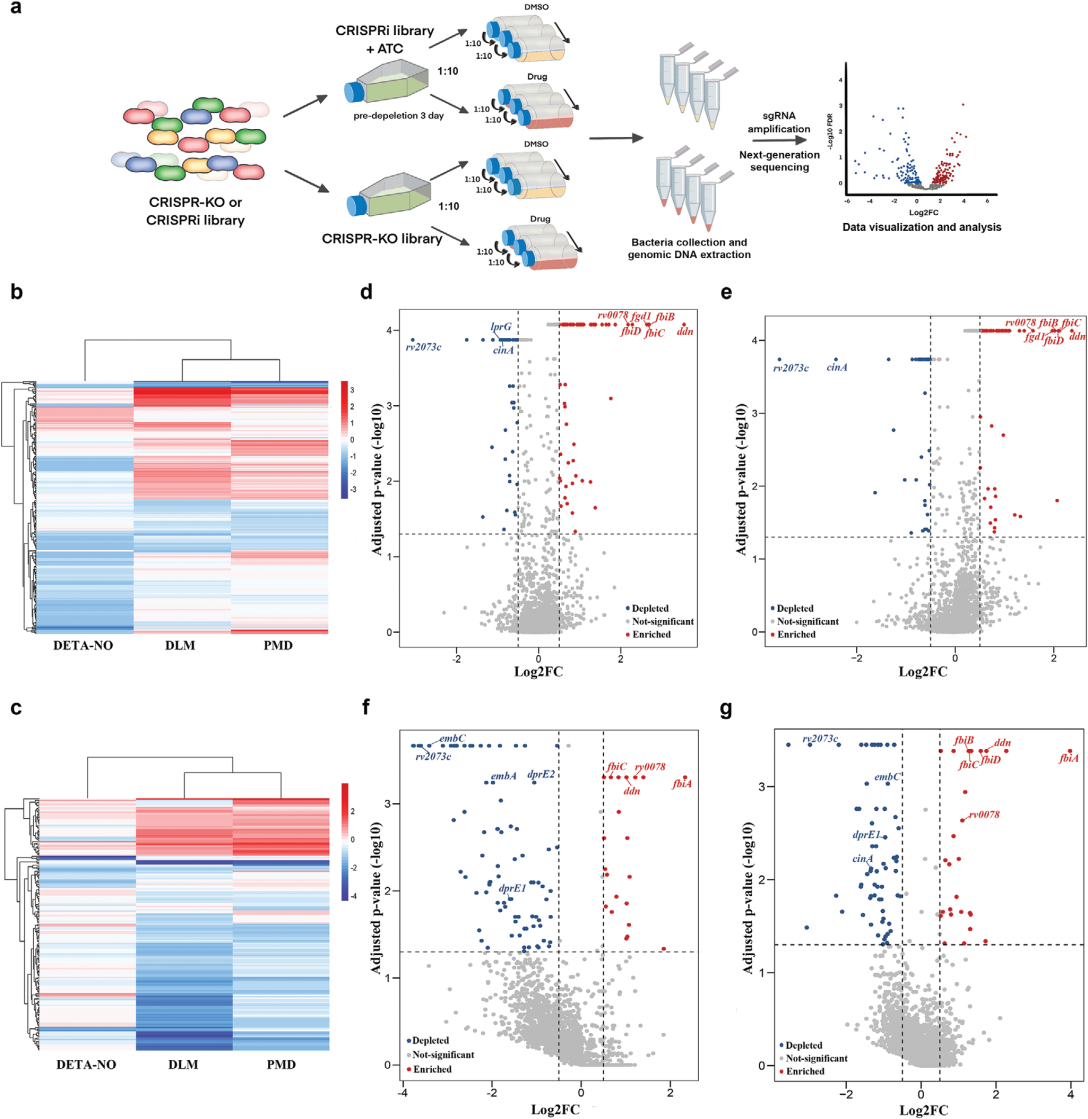
Combined CRISPR chemical‐genetic screening of DLM, PMD and DETA/NO. a) Combined CRISPR chemical‐genetic screening workflow. The CRISPRi and CRISPR‐KO libraries were treated with ≈1× MIC of DLM or PMD for 10 generations. b,c) Heatmaps representing identified genes in b) CRISPR‐KO and c) CRISPRi screening samples after treatment with DLM, PMD, or DETA/NO. Results are expressed as the mean value of two biological replicates. The color scale represents log2 fold‐changes, and the dendrogram shows hierarchical clustering of the genes based on Euclidean distance. d–g) Volcano plots showing the log2FC values and false discovery rates of each gene in the d,e) CRISPR‐KO and f,g) CRISPRi libraries after treatment with d,f) DLM or e,g) PMD.

The genetic determinants of Mtb sensitivity and resistance to DLM and PMD were analyzed by chemical‐genetic profiling at the genome scale using a combined CRISPR screening platform.^[^ ^30^ ^]^ This platform includes a CRISPR interference (CRISPRi) library and a CRISPR knock out (CRISPR‐KO) library, consisting of approximately 80 000 single guide RNAs (sgRNAs) targeting over 95% of the genes in the H37Ra Mtb genome. To repress target gene expression before treatment with DLM and PMD, the CRISPRi screening library was treated with anhydrotetracycline (ATc) for 3 d, after which both the CRISPRi and CRISPR‐KO libraries were treated with ≈1× minimum inhibitory concentration (MIC) of DLM and PMD for 10 generations (**Figure** **1**a). The sgRNAs from the libraries were amplified and analyzed by next generation sequencing. MAGeCK analysis showed that some of the genes recovered from CRISPR screening after treatment with PMD were identical to those recovered after treatment with DLM, whereas other genes were unique to each treatment (Figure 1b,c and Tables S1 and S2, Supporting Information). The most highly enriched genes were those involved in resistance to both PMD and DLM, including the *ddn*, *fgd1*, and *fbiA* genes (Figure 1d–g). Consistent with previous screening results, genes involved in cell envelope synthesis were associated with sensitization to PMD and DLM (Tables S1 and S2, Supporting Information).^[^ ^31^, ^32^ ^]^ The most depleted gene, *rv2073c*, and one of the most enriched genes, *rv0078*, were not identified during screening with other drugs.^[^ ^30^, ^31^, ^32^ ^]^ The *cinA* (*rv1901*) gene, which encodes an enzyme responsible for the cleavage of NAD‐PMD adducts,^[^ ^24^ ^]^ was recovered as a depleting gene; however, *dprE2*, a gene thought to be targeted by both the active adducts of PMD and DLM,^[^ ^17^ ^]^ was identified as a moderately‐sensitizing gene for DLM, but not for PMD. Only a few genes involved in mycolic acid synthesis were depleted in our screening (Tables S1 and S2, Supporting Information), but they were also depleted when Mtb were treated with other drugs,^[^ ^30^, ^31^, ^32^ ^]^ indicating they might not specifically affect the efficacy of DLM and PMD.

**Figure 2:**
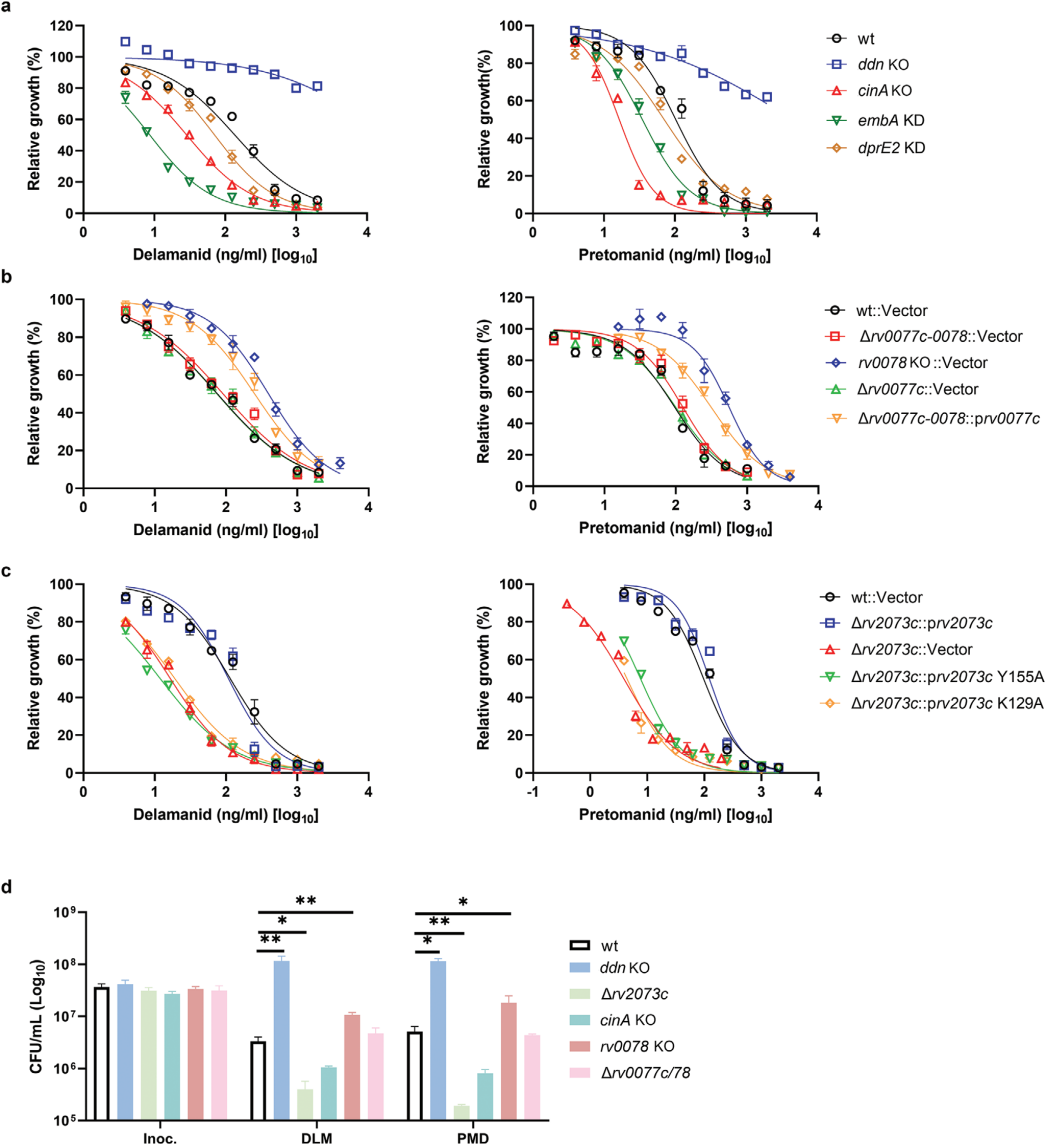
Validation of genetic screening results. a–c) Susceptibility of the indicated H37Ra CRISPRi knockdown (KD) and CRISPR‐KO strains to the antibiotics DLM and PMD. The MIC_50_ was defined as the concentration of compound that inhibited bacterial growth by 50%, and was analyzed by a nonlinear fit model in GraphPad Prism 9.0. Data are expressed as the mean ± SD of triplicate samples, and are representative of at least two independent experiments. d) Kill‐kinetic assays of the indicated strains. Bacteria were exposed to 10 µg mL^−1^ DLM or 10 µg mL^−1^ PMD for 3 d and cultured on agar plates to determine CFUs. Data are expressed as the mean ± SD of four biological replicates, and are representative of two independent experiments. Statistical significance was assessed by two‐way ANOVA with Dunnett's multiple comparison test. *p < 0.05, **p < 0.01 for comparisons between wild‐type and mutant strains.

To validate these screening results, Mtb mutants were constructed using the CRISPR‐KO or CRISPRi methods, and their drug susceptibilities were analyzed by determining the minimum inhibitory concentration that inhibited bacterial growth by 50% (MIC_50_), and/or kill‐kinetics assays. Consistent with previous results, knockdown of *embA*, which increased drug permeability,^[^ ^30^ ^]^ enhanced the susceptibility of Mtb to DLM and PMD (**Figure** **2**a). Mutation of *ddn* or *rv0078* increased drug resistance (Figure 2a,b,d, and Figure S1, Supporting Information), whereas mutation of *rv2073c* or *cinA* increased drug susceptibility (Figure 2a,c,d, and Figure S1, Supporting Information). Although DprE2 was suggested to be the drug target of the active adduct of DLM/PMD,^[^ ^17^, ^20^ ^]^ knockdown of *dprE2* using CRISPRi in Mtb did not significantly affect Mtb susceptibility to DLM and PMD (Figure 2a). Taken together, these findings validated the screening results, and suggested that some of the genetic determinants affecting efficacy of DLM and PMD were similar, whereas others were different.

To explore the role of nitric oxide (NO) in the bactericidal action of DLM and PMD, a diethylenetriamine/NO adduct (DETA/NO), which has been used to generate NO,^[^ ^33^, ^34^ ^]^ was used to mimic the pressure of NO generated during metabolism of DLM and PMD. CRISPR screening of Mtb following treatment with DETA/NO revealed that the most depleted genes were related to the iron acquisition pathway, with other depleted genes encoding iron–sulfur proteins (Tables S1 and S2, Supporting Information). NO might react with the iron–sulfur clusters of these proteins, causing irreversible damage.^[^ ^35^ ^]^ Only a few of the genes identified during this screening were identical to those detected by screening with PMD and DLM (Figure 1b,c and Figure S2, Supporting Information). In addition, addition of NO scavenger carboxy‐PTIO (C‐PTIO) did not alter the efficacy of PMD in Mtb in our experimental condition (Figure S3, Supporting Information). Together, these results indicated that the NO released during activation of DLM and PMD might not be primarily responsible for the activity of these drugs, at least under these screening conditions.

### 2.2 Metabolism of DLM and PMD in Mtb

**Figure 3:**
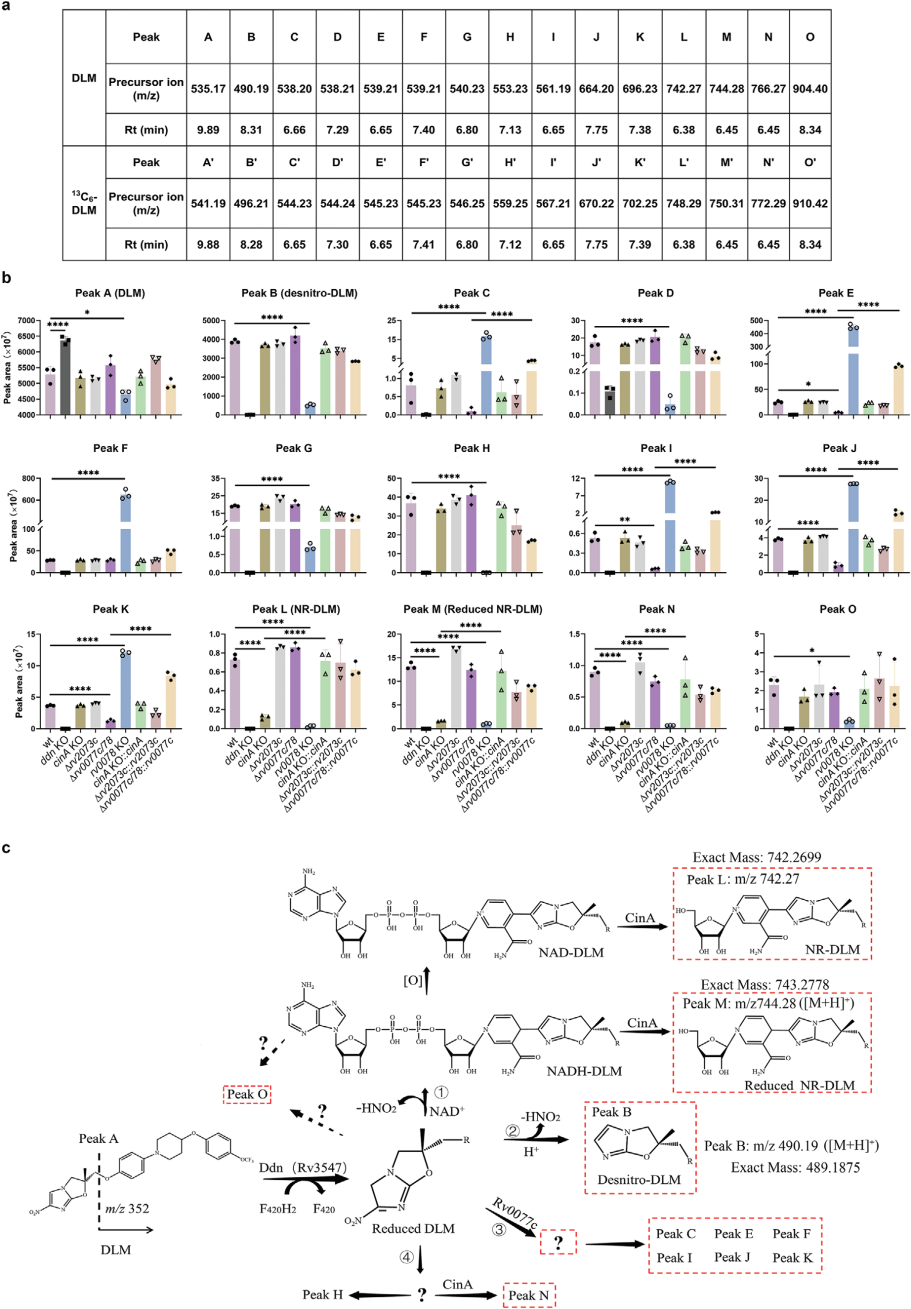
Identification of specific metabolites of DLM. a) Summary of precursor ions present in extracts of DLM‐ or ^13^C_6_‐DLM‐treated Mtb. Original precursor ion spectra of the extracts are shown in Figure S3 (Supporting Information). b) Relative abundance of the corresponding metabolites from the indicated strains in (a). Data are expressed as the mean ± SD of three cultures. Statistical significance was assessed by one‐way ANOVA with Tukey's multiple comparison test. *p < 0.05; ****p < 0.0001. c) Model of DLM metabolic pathways in mycobacteria based on the findings from this and previous studies.^[^ ^20^, ^24^ ^]^ Metabolites circled by dotted lines might not have antimycobacterial activity.^[^ ^9^, ^20^, ^24^ ^]^

Although several metabolites of DLM and PMD have been detected in Mtb,^[^ ^20^, ^24^ ^]^ the metabolic pathways of these two drugs are not clear. The metabolic pathways of DLM and PMD were therefore characterized by treating Mtb mutants showing different susceptibilities to DLM and PMD with DLM, isotope‐labeled DLM (^13^C_6_‐DLM)^[^ ^20^ ^]^ (Figure S4A, Supporting Information), or PMD. The resulting cellular extracts were subsequently analyzed by LC‐MS to identify DLM and PMD metabolites generated specifically by these mutants. The adducts of DLM and isotope‐labeled DLM would therefore be observed at the same chromatographic retention times, whereas isotope‐labeled DLM adducts would be 6 Da larger.^[^ ^20^ ^]^ Specific peaks detected in extracts isolated from DLM‐treated Mtb mutants are shown in **Figure** **3** and Figure S4B (Supporting Information). Most of these DLM adducts contain the specific 4‐(4‐[4‐trifluoromethoxyphenoxy]piperidin‐1‐yl)phenoxy group (m/z 352) (Figure 3 and Figure S5, Supporting Information), whereas two metabolites (with peaks of m/z 766 and 561) did not (Figure S5, Supporting Information), indicating that a moiety of the 4‐(4‐[4‐trifluoromethoxyphenoxy]piperidin‐1‐yl)phenoxy group may have reacted with other chemicals in these two compounds. The PMD adducts usually contain the specific 4‐trifluoromethoxybenzyl group (m/z 175) (Figures S6 and S7, Supporting Information), with the specific peaks detected in extracts isolated from PMD‐treated Mtb mutants being shown in Figure S6A (Supporting Information). The previously reported adducts of nicotinamide‐riboside (NR)‐DLM (peak L), reduced NR‐DLM (peak M), and peak J, were detected in the present study.^[^ ^20^ ^]^ By contrast, the NADH‐DLM, NAD‐DLM and NADH‐PMD adducts were not detected.^[^ ^20^, ^24^ ^]^

Deletion of *cinA* specifically reduced the peaks of NR‐DLM, and of reduced NR‐DLM (at m/z 742 and 744, respectively), in DLM‐treated Mtb (Figure 3b). These chemicals are suggested to be derivatives of NAD‐DLM and NADH‐DLM.^[^ ^20^ ^]^ Peaks consistent with NR‐PMD and reduced NR‐PMD (at m/z 567 and 569, respectively) were observed in PMD‐treated *Mtb*, with the intensities of these peaks also reduced in *cinA* mutants (Figure S6A, Supporting Information). Consistent with involvement of CinA in cleaving the corresponding NAD adducts in Mtb,^[^ ^24^ ^]^ the results of the present study suggest that CinA may recognize the pyrophosphate in NAD adducts, and cleave these adducts to produce NR derivatives (Figure 3c and Figure S6B, Supporting Information). Because mutations of *cinA* in Mtb increase bacterial susceptibility to DLM and PMD while reducing the levels of NR derivatives (Figure 3b and Figure S6A, Supporting Information), these results suggest that the NAD adducts, but not the NR derivatives, might be the antimycobacterial forms of DLM and PMD.^[^ ^20^ ^]^

*rv0078* encodes a transcriptional regulator that negatively regulates *rv0077c*.^[^ ^36^ ^]^ Deletion of both *rv0077c* and *rv0078* abolished drug resistance of *rv0078* mutant (Figure 2b), whereas high expression of Rv0077c in the strain bearing deletion of both *rv0077c* and *rv0078* partially rescued drug resistance (Figure 2b). These findings indicate that mutation in *rv0078* mediates resistance to PMD and DLM through regulation of *rv0077c*. The level of peak B (m/z 490), representing desnitro‐DLM (the major metabolite of DLM in DLM‐treated Mtb),^[^ ^9^ ^]^ was strongly decreased in the *rv0078* mutant, but not in the *rv0077c* and *rv0078* double mutant (Figure 3b). The levels of several other metabolites, including peaks D, G, H, L, M, N, and O, were also reduced significantly in the DLM‐treated *rv0078* mutant, but not in the *rv0077c* and *rv0078* double mutant (Figure 3b). In addition, several metabolites such as peak C, peak E, peak F, peak I, peak J, and peak K were significantly increased in the DLM‐treated *rv0078* mutant, but not in the *rv0077c* and *rv0078* double mutant (Figure 3b). Furthermore, high expression of Rv0077c rescued the phenotype of the *rv0077c* and *rv0078* double deletion mutant. Similar results were also observed for isotope‐labeled DLM or PMD‐treated Mtb strains (Figures S4B and S6A, Supporting Information). Taken together, these results suggest that *rv0077c* may encode an enzyme that catalyzes a reaction between the intermediates of DLM/PMD activated by Ddn and other unknown chemicals (Figure 3c and Figure S6B, Supporting Information). This, in turn, may lead to increased synthesis of some metabolites and reduced synthesis of NAD adducts and other related compounds (Figure 3c and Figure S6B, Supporting Information).

### 2.3 Rv2073c Is an Intrinsic Resistance Gene in Mtb That Is Specific for PMD and DLM

**Figure 4:**
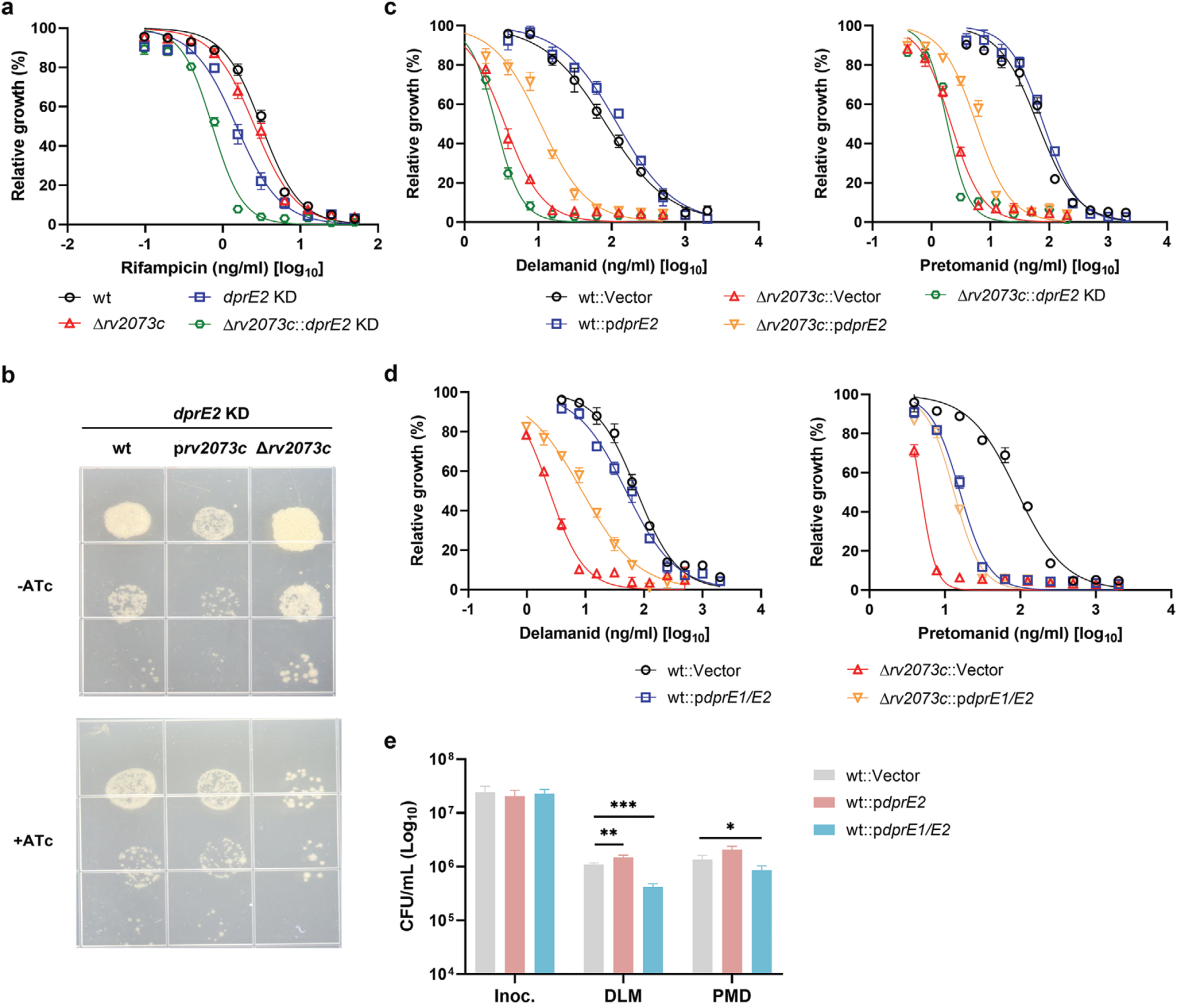
Rv2073c functions as an intrinsic gene conferring resistance to DLM and PMD by compensating for the role of DprE2 in Mtb. a) Susceptibility of the indicated Mtb H37Ra strains to RIF. b) Ten‐fold serial dilution spotting of Mtb strains of wild‐type, over‐expression of *rv2073c*, or *rv2073c* deletion with CRISPRi knocking down of DprE2. c,d) Susceptibility of the indicated Mtb H37Ra strains to DLM and PMD. The MIC_50_ was defined as the concentration of compound that inhibited bacterial growth by 50, and was analyzed by a nonlinear fit model in GraphPad Prism 9.0. Data are expressed as the mean ± SD of triplicate samples, and are representative of at least two independent experiments. e) Kill‐kinetic assays of the indicated strains. Bacteria were exposed to 10 µg mL^−1^ DLM or 10 µg mL^−1^ PMD for 3 d and cultured on agar plates to determine CFUs. Data are expressed as the mean ± SD of four biological replicates, and are representative of two independent experiments. Statistical significance was assessed by two‐way ANOVA with Dunnett's multiple comparison test. *p < 0.05, ***p < 0.001 for comparisons between wild‐type and overexpression strains.

Neither deletion nor over‐expression of *rv2073c* in Mtb affected the levels of metabolites of DLM/PMD (Figure 3b and Figure S4B, Supporting Information), suggesting that Rv2073c may not be involved directly in metabolism of DLM/PMD. To investigate the role of *rv2073c*, a strain bearing the *rv2073c* mutant, and a complementary strain, were tested for their susceptibility to various drugs. Consistent with this and previous screening results,^[^ ^30^, ^31^, ^32^ ^]^ deletion of *rv2073c* strongly affected bacterial susceptibility to PMD and DLM (Figure 2c), but not to other drugs (**Figure** **4**a and Figure S8A, Supporting Information), indicating that *rv2073c* is specifically responsible for resistance to PMD and DLM. The drug susceptibility phenotype of the *rv2073c* mutant could be complemented by a plasmid expressing wild‐type Rv2073c, but not Rv2073c harboring mutations in conserved amino acids (Figure 2c). Deletion of *rv2073c* did not affect the growth (Figure S8B, Supporting Information), lipid composition (Table S3, Supporting Information), cell metabolites (Table S4, Supporting Information), cell‐wall thickness (Figure S8C,D, Supporting Information), or cell permeability of Mtb (Figure S8E, Supporting Information) significantly, indicating that *rv2073c* might not affect drug susceptibility by interfering with uptake of PMD/DLM.

NCgl1429, an ortholog of Rv2073c in *Corynebacterium glutamicum*, functions in a manner similar to NCgl0186, an ortholog of DprE2.^[^ ^37^ ^]^ Over‐expression of NCgl1429 complements the defect in arabinogalactan resulting from deletion of NCgl0186 in *C. glutamicum*.^[^ ^37^ ^]^ To determine whether Rv2073c plays a role similar to that of DprE2 in Mtb, *dprE*2 in strains with high expression or mutation of *rv2073c* was depleted by CRISPRi. Over‐expression of Rv2073c reduced vulnerability of Mtb to DprE2, whereas deletion of *rv2073c* increased vulnerability to DprE2 (Figure 4b). Consistent with this result, deletion of *rv2073c* significantly affected lipid composition when DprE2 was depleted (Table S5, Supporting Information), whereas deletion of *rv2073c* did not significantly affect lipid composition in the wild type background (Table S3, Supporting Information). Taken together, these results indicated that Rv2073c can, at least partially, complement the activity of DprE2 in Mtb.

Over‐expression of DprE2 strongly increased resistance of *Mycobacterium bovis* bacillus Calmette‐Guérin (BCG) to DLM and PMD^[^ ^17^ ^]^ (Figure S9, Supporting Information), which supports the suggestion that DprE2 is the target of NAD adducts of DLM and PMD. However, over‐expression of DprE2 did not significantly affect the MIC_50_ of wild‐type Mtb (Figure 4c). The ortholog of *rv2073c* is absent from the genome of BCG^[^ ^37^ ^]^; therefore, we hypothesized that the presence or absence of Rv2073c is responsible for the differential DLM/PMD drug‐resistance phenotypes of Mtb and BCG overexpressing *dprE*2. Consistent with this hypothesis, over‐expression of DprE2 in the Mtb *rv2073c* mutant strain resulted in a phenotype similar to that of BCG following treatment with DLM/PMD (Figure 4c and Figure S9, Supporting Information). Depletion of *dprE*2 increased permeability of wild‐type Mtb and the *rv2073c* mutant strain (Figure S8E, Supporting Information), increasing their susceptibility to rifampicin (Figure 4a); however, depletion of *dprE*2 only slightly affected the MIC_50_ of wild‐type Mtb and the *rv2073c* mutant for DLM and PMD (Figures 2a and 4c). Depletion of dprE2 by CRISPRi in wild‐type Mtb and the *rv2073c* mutant was confirmed by quantitative reverse transcription PCR (RT‐qPCR) (Figure S10, Supporting Information). Taken together, these results indicated that DprE2 might not be the only target of DLM and PMD.

Over‐expression of the DprE1‐DprE2 complex increased the MIC_50_ of DLM by 3.7‐fold and PMD by 2.4‐fold against the *rv2073* mutant (Figure 4d), which is similar to over‐expression of DprE2 alone in the *rv2073c* mutant (Figure 4c). However, over‐expression of DprE1‐DprE2 in wild‐type Mtb results in a phenotype distinct from that of cells overexpressing DprE2 alone. Over‐expression of DprE1‐DprE2 in Mtb reduced the MIC_50_ of DLM by 1.3‐fold and that of PMD in wild‐type Mtb by fivefold (Figure 4d), while increasing the killing ability of PMD by 1.2‐fold and of DLM by 2.9‐fold (Figure 4e). These results indicate that these drugs inhibit growth and kill Mtb through more than one mechanism. In addition, the results also suggest that the DprE1‐DprE2 complex might potentiate the killing activity of DLM and PMD, which counteracts over‐expression of the drug target DprE2. LC‐MS analysis did not identify any new DLM metabolites in Mtb, and the amounts of most of DLM metabolites were not altered when the DprE1‐DprE2 complex was overexpressed (Figure S11, Supporting Information). However, the amounts of derivatives NAD‐DLM adducts (e.g., NR‐DLM and reduced NR‐DLM) were significantly increased following overexpression of DprE1‐DprE2 but not DprE2 in wild type Mtb and *rv2073c* deletion mutant (Figures S11 and S12, Supporting Information), indicating that the DprE1‐DprE2 complex could potentially facilitate synthesis of NAD adducts of DLM.

### 2.4 Mutation of Rv2073c Potentiates the Efficacy of PMD In Vivo

**Figure 5:**
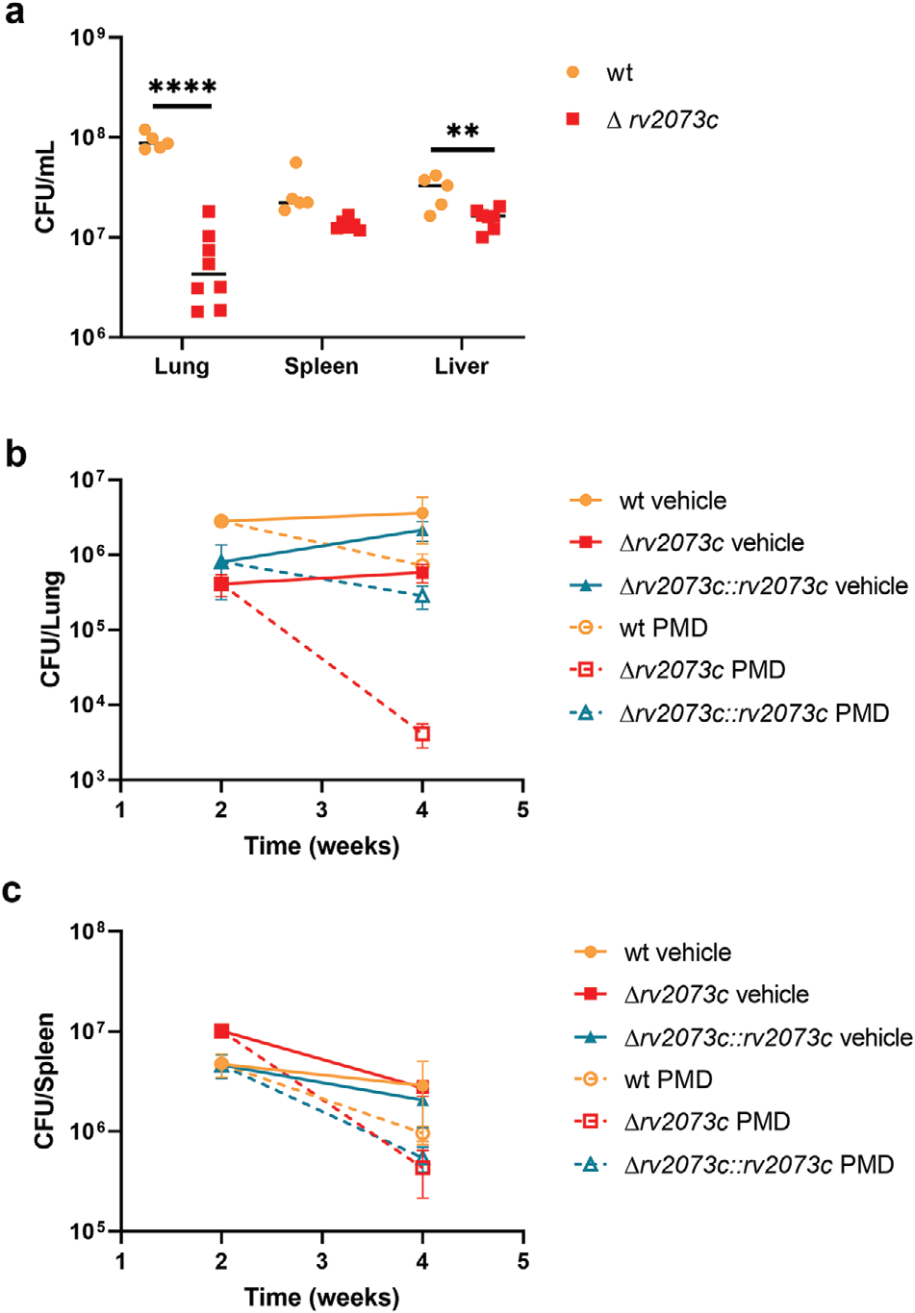
Deletion of *rv2073c* increases the efficacy of PMD in vivo. a) C57BL/6 mice were infected with wild‐type or Δ*rv2073c* Mtb using an infectious dose of 1.18 × 10^7^ and 1.54 × 10^7^ CFU, respectively. Bacterial burden (CFU) in the lungs, livers, and spleen was assessed after 4 weeks. Data are expressed as the mean ± SD of five (wild‐type) or eight (Δ*rv2073c*) replicates per experiment. Statistical significance was assessed by unpaired t‐tests. **p < 0.01; ****p < 0.0001. b,c) C57BL/6 mice were infected with wild‐type, Δ*rv2073c*, or the complemented strain using an infectious dose of 5.6 × 10^6^, 6.4 × 10^6^, and 5.9 × 10^6^ CFU, respectively. The infected mice were treated with PMD starting 2 weeks post‐infection. Bacterial burden (CFU) in the b) lungs and c) spleen was assessed 2‐ and 4‐weeks postinfection. Data are expressed as the mean ± SD of five replicates per experiment.

To test the role of Rv2073c mutation on the virulence of Mtb, and on the efficacy of the bicyclic nitroimidazole drugs in vivo, we infected mice with wild‐type Mtb or the *rv2073c* mutant by intravenous injection. Deletion of *rv2073c* slightly reduced virulence of Mtb, as assessed by colony forming assays of mouse lung, spleen, and liver tissue (**Figure** **5**a). Infection of these mice, followed 2 weeks later by treatment with PMD for 2 weeks, showed that the reduction in the number of colony‐forming units (CFU) was greater in mice infected with Mtb bearing mutated Rv2073c than in wild‐type Mtb, particularly in the lungs, where the CFU of the *rv2073c* mutant declined by over 100‐fold, while the CFU of wild‐type Mtb declined by about fivefold (Figure 5b). These findings suggest that Rv2073c may be a drug target that potentiates the efficacy of PMD, thereby shortening the duration of TB chemotherapy.

## 3 Discussion

PMD and DLM, which belong to a novel class of antimicrobial drugs used to treat TB, kill Mtb upon activation by Ddn^[^ ^12^ ^]^; however, the active adducts, their metabolism, and the mechanisms by which they kill Mtb are not well known. A better understanding of the genetic factors that influence the efficacy of these drugs could help to answer these questions and facilitate design of synergistic drug combinations. The present study utilized established CRISPR‐KO and CRISPRi libraries for genome‐scale screening to identify determinants of sensitivity and resistance to PMD and DLM. Dozens of genes were identified as determinants of responses to both drugs, whereas others were unique to each; these findings are consistent with previous findings that DLM and PMD share many similarities but also have unique features.^[^ ^12^ ^]^ The findings presented herein provide a global overview of the genes and pathways that influence the activity of these drugs, and allow for the design of drug combinations that potentiate the activity of PMD and DLM, as well as detecting emergence of drug resistance.

Screening identified the *rv2073c* gene as being the most depleted, with mutations in this gene not only reducing the MIC_50_ values for PMD and DLM, but also increasing their killing efficacy. Rv2073c is responsible for intrinsic resistance to PMD and DLM, but is not involved in resistance to other drugs. Rv2073c might affect the efficacy of DLM/PMD by regulating vulnerability of the drug target DprE2. Similar to the function of the corresponding ortholog in *C. glutamicum*, Rv2073c can complement, at least partially, the function of DprE2 in Mtb, suggesting that Rv2073c might compensate for functional repression of DprE2 by DLM/PMD adducts. Alternatively, Rv2073c may bind to NAD‐DLM/PMD adducts as DprE2^[^ ^17^ ^]^ and thus function as a natural sink for these toxic compounds.

Interestingly, the screening protocol conducted in this study identified a new mechanism of resistance to PMD and DLM. A mutation in *rv0078*, a transcriptional repressor of Rv0077c,^[^ ^36^ ^]^ strongly increased resistance to PMD and DLM in a Rv0077c‐dependent manner. Rv0077c may be involved in metabolism of reduced intermediates of DLM and PMD (Figure 3c), thereby preventing synthesis of the active anti‐TB chemicals NAD‐DLM and NAD‐PMD. Mutations of *rv0078* increases expression of Rv0077c, thereby increasing resistance to PMD and DLM. Sequence analysis showed that 3.3% of 51229 sequenced Mtb clinically isolates have nonsynonymous mutations (Table S6, Supporting Information). Because mutations in *rv0078* might limit the clinical efficacy of PMD and DLM, such mutations should be investigated and monitored in patients treated with these drugs.

NAD+ may bind covalently to reduced DLM and PMD to form NAD adducts with antimycobacterial activity.^[^ ^20^, ^24^ ^]^ Comprehensive LC‐MS analysis of extracts of DLM‐ or PMD‐treated Mtb suggested possible metabolic pathways for activated DLM and PMD in Mtb (Figure 3c and Figure S6B, Supporting Information). Ddn‐reduced DLM or PMD are converted to 1) desnitro‐DLM/PMD by reacting with water, 2) NADH‐DLM/PMD by reacting with NAD+ or 3) metabolites by reacting with unknown chemical catalyzed by Rv0077c (Figure 3c and Figure S6B, Supporting Information). Although the detailed mechanism(s) by which Rv0077c catalyzes the reduction of DLM or PMD remain undetermined, several specific metabolites that are dependent on the presence of Rv0077c were identified in Mtb. In addition, the pyrophosphate moiety present in these NAD adducts may be recognized by the pyrophosphatase domain of CinA, followed by degradation of CinA to yield reduced NR‐DLM/PMD and NR‐DLM, respectively (Figure 3c and Figure S6B, Supporting Information).

NAD+ or NADH is required to convert Ddn‐activated PMD to an active adduct that can inhibit DprE2 activity;^[^ ^17^ ^]^ however, neither NAD+ nor NADH is required to inhibit DprE2 activity if DprE1‐DprE2 is present during PMD‐activation by Ddn in vitro.^[^ ^17^ ^]^ DprE1‐DprE2 complexes might provide an endogenous source of the cofactor NAD (NADH) for formation of activated adducts.^[^ ^17^ ^]^ The present study found that over‐expression of DprE1‐DprE2 led to increased production of the metabolites of NAD‐DLM when Mtb was treated with DLM, suggesting that DprE1‐DprE2 might facilitate the conversion of DLM/PMD to their active NAD adducts. This is further supported by results showing that over‐expression of DprE1‐DprE2 in Mtb increased susceptibility to DLM/PMD (Figure 4d). In addition, inhibiting DprE2 activity may not be the sole mechanism by which DLM/PMD kill Mtb. This is supported by the follow findings: 1) depleting the target DprE2 in Mtb did not increase susceptibility to DLM/PMD; and 2) over‐expression of DprE2 in Mtb did not increase resistance of wild‐type Mtb to DLM/PMD. Other mechanisms contributing to the antimycobacterial activity of DLM and PMD still need to be investigated.

In conclusion, the present study employed CRISPR screening to identify specific genes that affect the efficacy of PMD and DLM against Mtb. It is important to note that our screening was conducted using the lab strain H37Ra. While some of the screened genes were validated in the Mtb H37Rv strain, it is worth acknowledging that screening outcomes might vary in clinical strains. Rv2073c was identified as a major mediator of intrinsic resistance to these drugs. Designing an inhibitor to target this protein to potentiate DLM and PMD activity may increase the efficacy of treatments for drug‐resistant TB. In addition, characterization of the metabolic pathways of PMD, DLM, and their active adducts might help development of new derivatives of PMD and DLM that can overcome intrinsic resistance pathways in Mtb.

## 4 Experimental Section

### Strains and Plasmids

*M. tuberculosis* strains H37Ra, H37Rv, *M. bovis* BCG, and their derivatives were cultured at 37 °C in Middlebrook 7H9 broth (Difco) supplemented with 0.05% Tween 80, 0.2% glycerol, and oleic acid‐albumin‐dextrose‐catalase (OADC; Becton Dickinson), or on 7H10 plates supplemented with 0.5% glycerol, OADC, and appropriate antibiotic (kanamycin 25 µg mL^−1^; hygromycin 50 µg mL^−1^; or zeocin 50 µg mL^−1^). The attenuated Mtb strain H37Ra was used as the wild‐type for this study. *M. tb* strain H37Rv and its derivatives were cultured in a BSL3 facility. Bacteria were treated with 100 ng mL^−1^ ATc as indicated. CRISPRi and CRISPR‐KO strains were constructed as described previously.^[^ ^30^ ^]^

The complemented strains were generated by introducing a copy of wild‐type *rv2073c*, *rv0077c*, or *cinA* into the *Mtb* genome at the attL5 attachment site using integrated plasmids. Complementation plasmids were generated by introducing the *rv2073c*, *rv0077c*, or *cinA* open reading frame (ORF) sequence into an integrated plasmid, pYC2364, under the control of a TetR‐regulated promoter. Complementation strains expressing *rv2073c* harboring point mutations (Y155A; K129A) were obtained using the same approach. The plasmid overexpressing DprE1‐DprE2 was constructed by introducing the *dprE1‐dprE2* operon and its native promoter (470 bp upstream of the coding region) into pSE100, resulting in plasmid pYC3169. The plasmid overexpressing DprE2 was constructed by incorporating the ORF of *dprE2* into the plasmid pYC601 under the control of a TetR‐regulated promoter,^[^ ^38^ ^]^ resulting in plasmid pYC3170. The primers used in this study are described in Table S7 (Supporting Information). Strains constructed in this work are listed in Table S8 (Supporting Information).

### Combined CRISPR Chemical‐Genetic Screening

Each CRISPRi or CRISPR‐KO library stock containing ≈3 × 10^8^ cells was incubated in Middlebrook 7H9 medium for 7 d at 37 °C to allow recovery. The recovered cultures were further diluted in 7H9 medium (100 ng mL^−1^ ATc was added to the CRISPRi library) at a starting OD_600_ of 0.1, and the cultures were grown to an OD_600_ of 1.0. The CRISPRi and CRISPR‐KO library starter cultures were inoculated at an OD_600_ of 0.1 into 30 mL of 7H9 medium supplemented with the indicated drug (DLM, PMD, or DETA/NO in DMSO) or DMSO alone, with 100 ng mL^−1^ ATc added to the CRISPRi cultures. The cultures were incubated at 37 °C until the OD_600_ of the antibiotic‐free control culture reached ≈1.0. Combined CRISPR screenings were performed in roller bottles through three rounds of 1:10 culture dilutions to standardize the number of outgrowth generations between libraries at approximately 10 generations. Each experiment included two replicates.

### Extraction of Genomic DNA and Library Preparation for Illumina Sequencing

After drug treatment, the cultures were pelleted by centrifugation (10 min at 3700 × *g*) and washed with phosphate‐buffered saline (PBS); pellets were then frozen until processing. Genomic DNA was isolated from bacterial pellets using the cetyltrimethylammonium bromide (CTAB)‐lysozyme method, as described.^[^ ^39^ ^]^ Briefly, 3 × 10^9^ Mtb cells were resuspended in 500 µL of GTE buffer (50 × 10^−3^ m glucose, 25 × 10^−3^ m Tris‐HCl pH 8, 10 × 10^−3^ m EDTA) containing 20 mg mL^−1^ lysozyme, and incubated at 37 °C overnight. A 100 µL aliquot of SDS and 50 µL of 10 mg mL^−1^ proteinase K were added to each sample. Samples were then incubated at 65 °C for 30 min. Following addition of 200 µL 5 m NaCl and 160 µL of CTAB to each sample, they were incubated at 65 °C for another 30 min. Finally, an equal volume (≈1 mL) 24:1 (v/v) chloroform/isoamyl alcohol was added to each sample and mixed vigorously prior to microcentrifugation for 5 min. A 900 µL aliquot of the aqueous layer of each sample was transferred to a fresh 2 mL microcentrifuge tube, and each sample was treated with 25 µg RNase A for 30 min at 37 °C, followed by extraction with 25:24:1 (v/v) phenol/chloroform/isoamyl alcohol. Genomic DNA was precipitated from the aqueous layer after adding 80 µL (0.1 vol) 3 m sodium acetate and 560 µL (0.7 vol) isopropanol. Finally, each DNA sample was washed twice with 750 µL of 70% ethanol and resuspended in RNase‐free water.

Next‐generation sequencing was performed as described previously.^[^ ^30^ ^]^ Genomic DNA was used as a template for PCR amplification of the sgRNA‐encoding region. Each 50 µL reaction solution contained 50 ng of template and I‐5 2X Hi‐Fi PCR Master Mix (MCLAB), with eight reactions tested per library. The CRISPR‐KO library was subjected to two‐step PCR to amplify the sgRNA barcodes, as most escapers from genome editing were found to carry large fragment deletions in Sth1 Cas9. The first PCR amplified a 5000 bp fragment containing the most commonly retained Cas9 sequence plus the sgRNA region. Each 25 µL reaction solution contained 50 ng of template and DreamTaq Green PCR Master MIX (Thermo Scientific; #K1081), with eight reactions tested per library. The PCR products were subjected to 0.7% polyacrylamide gel electrophoresis and purified using QIAquick Gel Extraction kits (Qiagen; # 28706). In the second PCR amplification, the region of library sgRNAs was amplified as described previously. The resulting 242‐bp PCR fragments were sent to GENEWIZ, Inc. (Suzhou, China) for amplicon sequencing. Sequencing results were analyzed using the MAGeCK robust rank analysis method.^[^ ^40^ ^]^

### In Vitro Antibiotic Susceptibility Testing

The MICs of compounds against various Mtb strains were determined using the broth microdilution technique, as described previously.^[^ ^30^ ^]^ Compounds dissolved in 90% DMSO were serially diluted (twofold) in 96‐well clear plates (Falcon; #3072). Bacteria were grown to mid‐log phase and adjusted to an OD_600_ of 0.04. A 100 µL aliquot of each culture was added to each well of 96‐well plates containing drugs and incubated for 7–12 d. OD_600_ values were recorded using an EnVision multimode microplate reader (PerkinElmer), and MIC_50_ curves were plotted using GraphPad Prism 9 software. CRISPRi strains were growth‐synchronized and pre‐depleted in the presence of ATc (100 ng mL^−1^) for 4 d prior to MIC analysis. Each experiment was performed twice independently, with three biological replicates per experiment.

For the kill‐kinetics assays, bacteria were grown to an OD_600_ of 1.0 in Middlebrook 7H9 medium at 37 °C, with 100 ng mL^−1^ ATc added to the CRISPRi cultures for pre‐depletion, followed by back‐diluting to a starting OD_600_ of 0.1. DLM and PMD were added at final concentrations of 10 µg mL^−1^ each. At the indicated time points, cultures were washed twice with PBS supplemented with 0.05% Tween 80, serially diluted, and plated onto 7H10 agar plates. The plates were incubated for 3 weeks at 37 °C, and the number of colonies was counted. Each kill‐kinetics assay was performed twice independently, with four biological replicates per experiment.

### Preparation and Detection of DLM and PMD Metabolites

DLM and PMD metabolites from cellular component extracts of drug‐treated *Mtb* strains were prepared as described, with minor modifications.^[^ ^20^, ^24^ ^]^ Briefly, wild‐type and mutant Mtb strains were grown to an OD_600_ of 0.8, washed with PBS supplemented with 0.05% Tween 80, and resuspended in 7H9 medium (0.2 vol) containing 50 µg mL^−1^ DLM, ^13^C_6_‐DLM, or PMD. After 4 h, each bacterial suspension was mixed with a twofold volume of acetonitrile. Each supernatant was transferred to a fresh centrifuge tube, to which a 0.5 volume of 2:1 (v/v) chloroform/methanol was added. The samples were mixed vigorously and centrifuged at 4000 × *g* for 10 min. The metabolite extracts were collected in the chloroform phase. Samples were stored at −80 °C until analysis by LC‐MS/MS. The extracts were analyzed by Shanghai Applied Protein Technology Co., Ltd., according to previously published chromatography methods.^[^ ^20^, ^24^ ^]^

### Total RNA Extraction and RT‐qPCR

RNA was isolated from mycobacterial cells using the RNeasy Mini Kit (Qiagen). Briefly, ≈6 OD_600_ units of bacteria were collected and resuspended in 1 mL of Qiagen RNA protect reagent. The cell suspension was then incubated for 1 h at room temperature. Following this, the cell suspension was pelleted by centrifugation, resuspended in 1 mL Qiagen buffer RLT and lysed by zirconium bead beating (MP Biomedicals; 116911050). Subsequently, approximately 750 µL of processed liquid was transferred to a new 2 mL microcentrifuge tube. 525 µL of absolute ethanol was added to each sample and the samples were then transferred to Qiagen RNeasy mini columns. Total RNA was purified according to Qiagen RNeasy kit manufacturer's instructions. After RNA cleanup and concentration, 1 µg RNA per sample was reverse transcribed into complementary DNA (cDNA) using the HiScript II RT SuperMix for qPCR (Vazyme). Next, knockdown of the targets was quantified by SYBR green dye‐based quantitative real‐time PCR (Vazyme; Q712‐02) on a CFX96 machine (Bio‐Rad). The relative expression of the target gene was normalized to *sigA* (*rv2703*) and quantified by the ΔΔCt algorithm. 

### Spotting Assay

The plasmid for knockdown of *dprE2* by CRISPRi was transformed to wild‐type, wild‐type overexpressing *rv2073c*, and *rv2073c* mutant, respectively. At least three transformants were picked and placed into a tube containing 100 µL 7H9 medium and mixed by vortex. Cultures were then serially diluted 10‐fold and spotted on plates with or without ATc. Plates were incubated at 37 °C for 21 d before imaging.

### Transmission Electron Microscopy

*Rv2073c* mutants and wild‐type strains were grown to an OD_600_ of about 1.0. The cells were then pelleted by centrifugation at 4000 × *g* at room temperature for 10 min and resuspended in electron microscope fixation solution (G1102, Servicebio, Wuhan, China). The cells were fixed at 4 °C for 24 h and washed three times with 0.1 m phosphate buffer (PB, pH 7.4) to remove fixatives. The cell pellets were suspended in 1% agarose solution, fixed with 1% osmium acid in the dark at room temperature for 2 h, and washed three times in PB. The samples were subsequently dehydrated at room temperature, followed by embedding in resin and slicing. The sections were stained in 2% uranium acetate saturated alcohol solution for 8 min, washed, and incubated overnight. Images were captured and analyzed under a transmission electron microscope (HT7800, HITACHI).

### Cell‐Wall Permeability Assay

Cell‐wall permeability was determined by measuring intracellular accumulation of ethidium bromide, as described previously.^[^ ^30^ ^]^ Briefly, Mtb strains were grown to an OD_600_ of 0.6–0.8 in Middlebrook 7H9 medium at 37 °C. The cultures were centrifuged at 3000 × *g* for 10 min and the supernatants were discarded. The pellets were washed once in PBS with 0.05% Tween 80 and adjusted to an OD_600_ of 0.8 in PBS supplemented with 0.4% glucose. Aliquots (100 µL) of bacterial suspension were added to each well of a black 96‐well plate (Costar), and an equal volume of 2 µg mL^−1^ EtBr in PBS containing 0.4% glucose was added to each well. EtBr fluorescence was measured every 90 s for 60 min at 37 °C in a multifunctional microplate reader (Tecan Infinite 200 pro) at an excitation wavelength of 530 nm and an emission wavelength of 590 nm.

### Lipidomics Analysis

Total lipids were extracted using the MTBE method.^[^ ^41^ ^]^ Briefly, Mtb strains were grown in 7H9 medium to an OD_600_ of 1.0. Cells were harvested by centrifuging 10 mL culture aliquots, followed by washing twice in PBS/0.05% Tween 80. The pellets were accurately measured and spiked with appropriate amounts of internal lipid standards, then homogenized with 100 µL water and 240 µL methanol. An 800 µL aliquot of MTBE was added to each sample and the mixture was sonicated for 20 min at 4 °C, followed by incubation for 30 min at room temperature. The samples were centrifuged at 14000× *g* for 15 min at 10 °C, and the upper layer of each was withdrawn and dried under nitrogen. The lipid extracts were redissolved in 200 µL of 90% isopropanol/acetonitrile and centrifuged at 14000× *g* for 15 min. The lipid extracts were decanted, and the cell pellets were subjected to an additional extraction with MTBE/methanol/water (10:3:2.5, vol/vol/vol). The extracts were pooled and dried under nitrogen gas. Lipidomics analysis was performed by Shanghai Applied Protein Technology Co., Ltd. After UPLC‐MS/MS analyses, the raw data were imported into the LipidSearch (Thermo, CA) for peak detection, alignment and identification. The lipids were identified by MS/MS fragments and by querying each m/z in the mycobacteria‐specific database MycoMass at a 5 ppm mass window with TraceFinder software.^[^ ^42^ ^]^ The preprocessing results generated a data matrix that consisted of the lipid class, retention time (RT), mass‐to‐charge ratio (m/z) values, and peak intensity. Perform variance analysis on the matrix file after data preprocessing. The selection of significantly different metabolites was determined based on the × 10^−3^ mariable importance in the projection (VIP) obtained by the OPLS‐DA model and the p‐value of student's t test, and the metabolites with VIP>1, *p* < 0.05 were significantly different metabolites.

### Metabolomics Analysis

Six independent cultures of each analyzed strain were grown in 7H9 medium to an OD_600_ of ≈1.0. Fifty OD equivalents per replicate were processed by chloroform‐methanol extraction.^[^ ^42^ ^]^ Metabolomic profiling was performed by the Shanghai Majorbio Bio‐pharm Technology Co., Ltd. (Shanghai, China). Raw data analysis, including peak extraction, baseline adjustment, deconvolution, alignment, and integration, was performed using Chroma TOF (v.4.3x, LECO) software.

### SNPs Calling

The raw WGS read data for the 51,229 *M. tuberculosis* isolates were downloaded from the NCBI database.^[^ ^43^ ^]^ The SNPs of *rv0078* were analyzed using a previously validated pipeline.^[^ ^44^ ^]^

### Mouse Infection

Mouse experiments to assess the virulence of the Δ*rv2073c* mutant and the impact of PMD on Mtb infection were approved by the Committee on the Use and Management of Laboratory Animals, Institute of Medical Laboratory Animals, Chinese Academy of Medical Sciences, Beijing (Approval No. ZLJ22001). This research was performed in accordance with the Guide for the Care and Use of Laboratory Animals. Animal rearing and experimentation were carried out in the Grade 3 Laboratory of Biosafety, Institute of Medical Laboratory Animals, Chinese Academy of Medical Sciences (ABSL3‐059). Briefly, 8 week old C57BL/6 mice were challenged intravenously with *M. tuberculosis* H37Rv in 200 µL of PBS. The Infection dose was determined by plating dilutions on 7H10 agar. The mice were housed in plastic cages (5 per cage) with free access to drinking water and a pellet diet, under controlled humidity (50 ± 10%), light (12/12 h light/dark cycle), and temperature (23 ± 2 °C) conditions in an Animal Biosafety Level 3 (ABSL‐3) facility. Beginning 14 d after infection, PMD (50 mg kg^−1^) was administered by gavage 5 d per week for 2 weeks. PMD was suspended in a cyclodextrin micelle (CM‐2) formulation containing 10% hydroxypropyl‐β‐cyclodextrin and 10% lecithin. At the indicated time points, the mice were sacrificed and spleen, liver, and lungs were isolated and homogenized. CFUs were determined by plating dilutions on 7H10 agar.

## Conflict of Interest

The authors declare no conflict of interest.

## Author Contributions

M.‐Y.Y., H.L., Y.‐M.Q., S.‐S.L., and D.Z. contributed equally to this work. Y.‐C.S., M.‐Y.Y., J.L., and Y.P. designed the project. M.‐Y.Y., H.L., Y.‐M.Q., S.‐S.L., and L.Z. performed the experiments. M.‐Y.Y., Y.‐M.Q., D.Z., X.‐P.G., Z.W., W.L., and J.Y. analyzed data. Y.‐C.S. and M.‐Y.Y. wrote the manuscript.

## Supporting information

Supporting Information

Supporting Table 1

Supporting Table 2

Supporting Table 3

Supporting Table 4

Supporting Table 5

Supporting Table 6

## Data Availability

The data that support the findings of this study are available in the Supporting Information of this article.
