# Supplementary material for: CRISPR Screening and Comparative LC‐MS Analysis Identify Genes Mediating Efficacy of Delamanid and Pretomanid against Mycobacterium tuberculosis
Source: Adv Sci (Weinh). 2024 Aug 20;11(39):2400176. doi: 10.1002/advs.202400176 (PMC11497083; doi:10.1002/advs.202400176)
Supplement: Supplementary file 1 — Supporting Information [file ADVS-11-2400176-s004.pdf]

ADVANCED SCIENCE
Open Access

Supporting Information

for *Adv. Sci.*, DOI 10.1002/advs.202400176

# CRISPR Screening and Comparative LC-MS Analysis Identify Genes Mediating Efficacy of Delamanid and Pretomanid against *Mycobacterium tuberculosis*

*Mei-Yi Yan, Haifeng Li, Yun-Mo Qu, Si-Shang Li, Dandan Zheng, Xiao-Peng Guo, Zhaojun Wu, Jie Lu, Yu Pang, Weimin Li, Jian Yang, Lingjun Zhan* and Yi-Cheng Sun**

## Supplementary Information

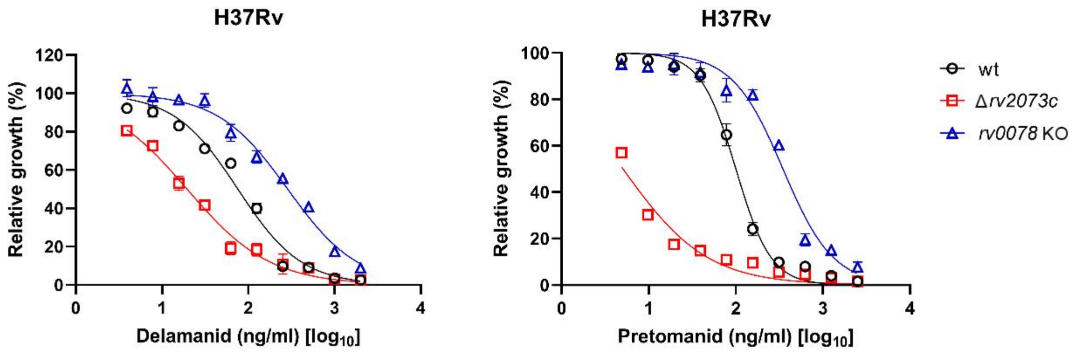


**Fig S1. Validation of screened genes in H37Rv by measuring MICs.** Susceptibility of the indicated H37Rv wild-type strain, *rv2073c* mutant strain and *rv0078* knockout strain to DLM and PMD. The $MIC_{50}$ was defined as the concentration of compound that inhibited bacterial growth by 50%, and was analyzed by a nonlinear fit model in GraphPad Prism 9.0. Data are expressed as the mean ± SD of triplicate samples, and are representative of at least two independent experiments.

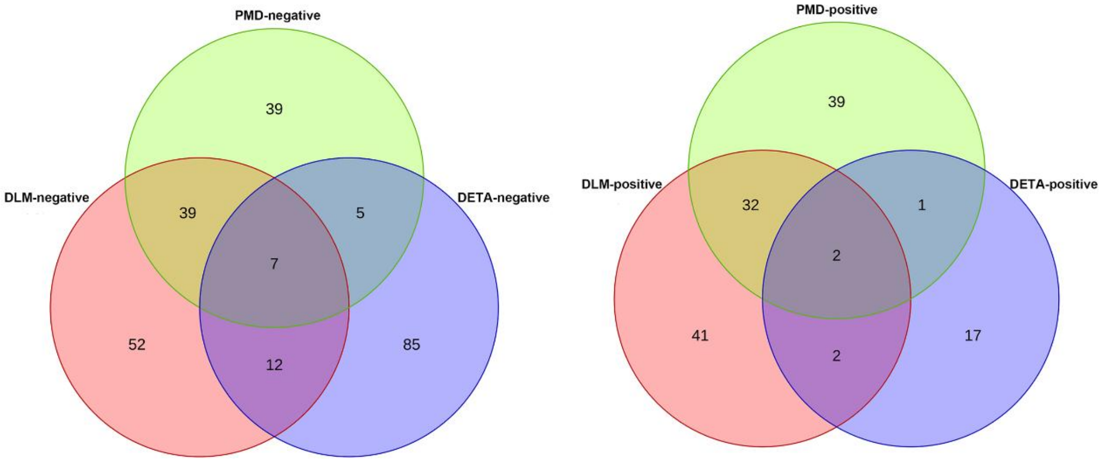


**Fig S2. Venn diagram comparing positive and negative genes identified by screening of CRISPRi and CRISPR-KO libraries following treatment with DLM, PMD, or DETA/NO.**

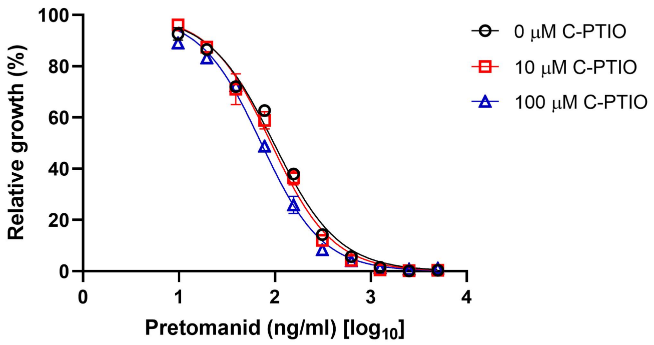


**Fig S3. PMD-induced growth inhibition of Mtb H37Ra in the absence or presence of indicated concentration of Carboxy-PTIO (C-PTIO).** The $MIC_{50}$ was defined as the concentration of compound that inhibited bacterial growth by 50%, and was analyzed by a nonlinear fit model in GraphPad Prism 9.0. Data are expressed as the mean ± SD of triplicate samples, and are representative of at least two independent experiments.

A

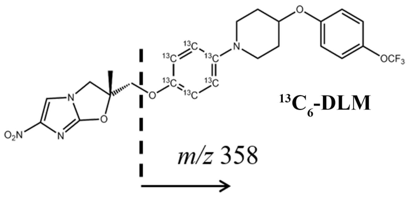


B

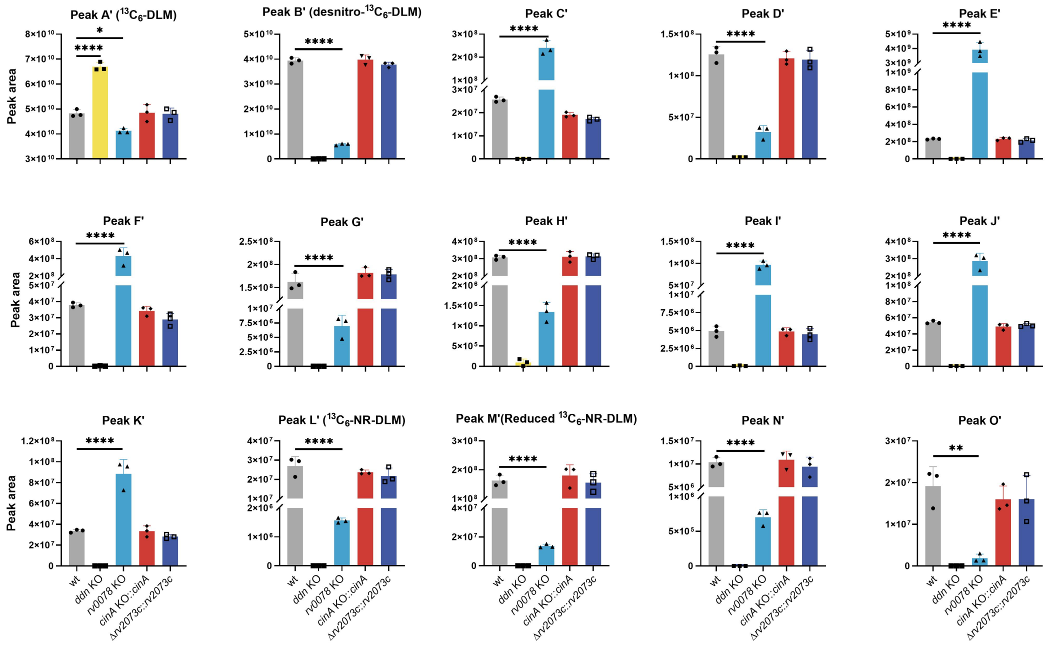


**Fig S4. Identification of specific metabolites of DLM. (A)** Chemical structure of $^{13}C_6$-DLM. **(B)** Quantification of the relative abundance of the corresponding metabolites from the $^{13}C_6$-DLM-treated Mtb mutants shown in Figure 3a. Data are expressed as the mean ± SD of three independent cultures. Statistical significance was assessed by one-way ANOVA with Tukey's multiple comparison test. * $P < 0.05$; ** $P < 0.01$; **** $P < 0.0001$.

A

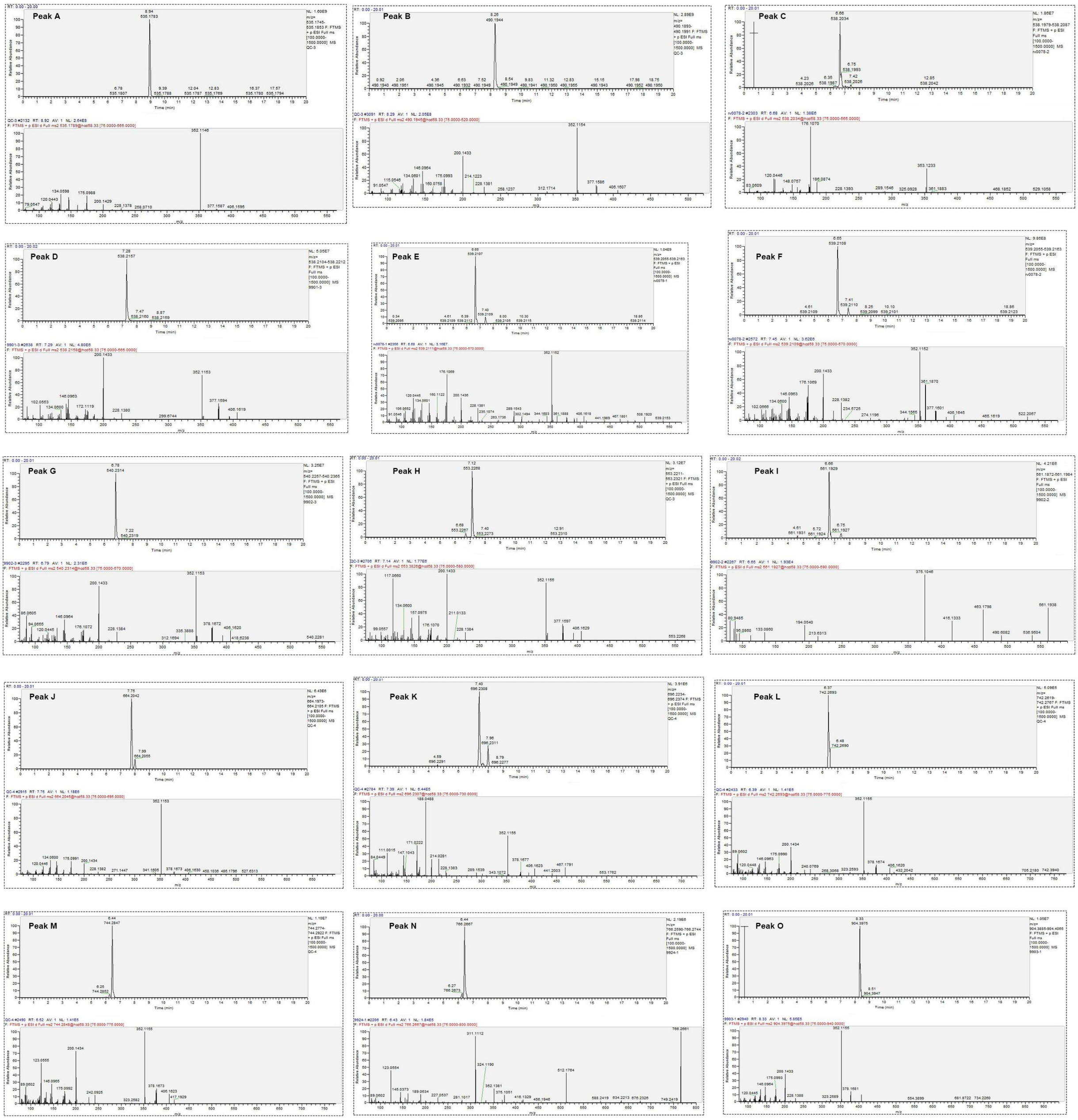
Peak A
Peak B
Peak C
Peak D
Peak E
Peak F
Peak G
Peak H
Peak I
Peak J
Peak K
Peak L
Peak M
Peak N
Peak O

**B**

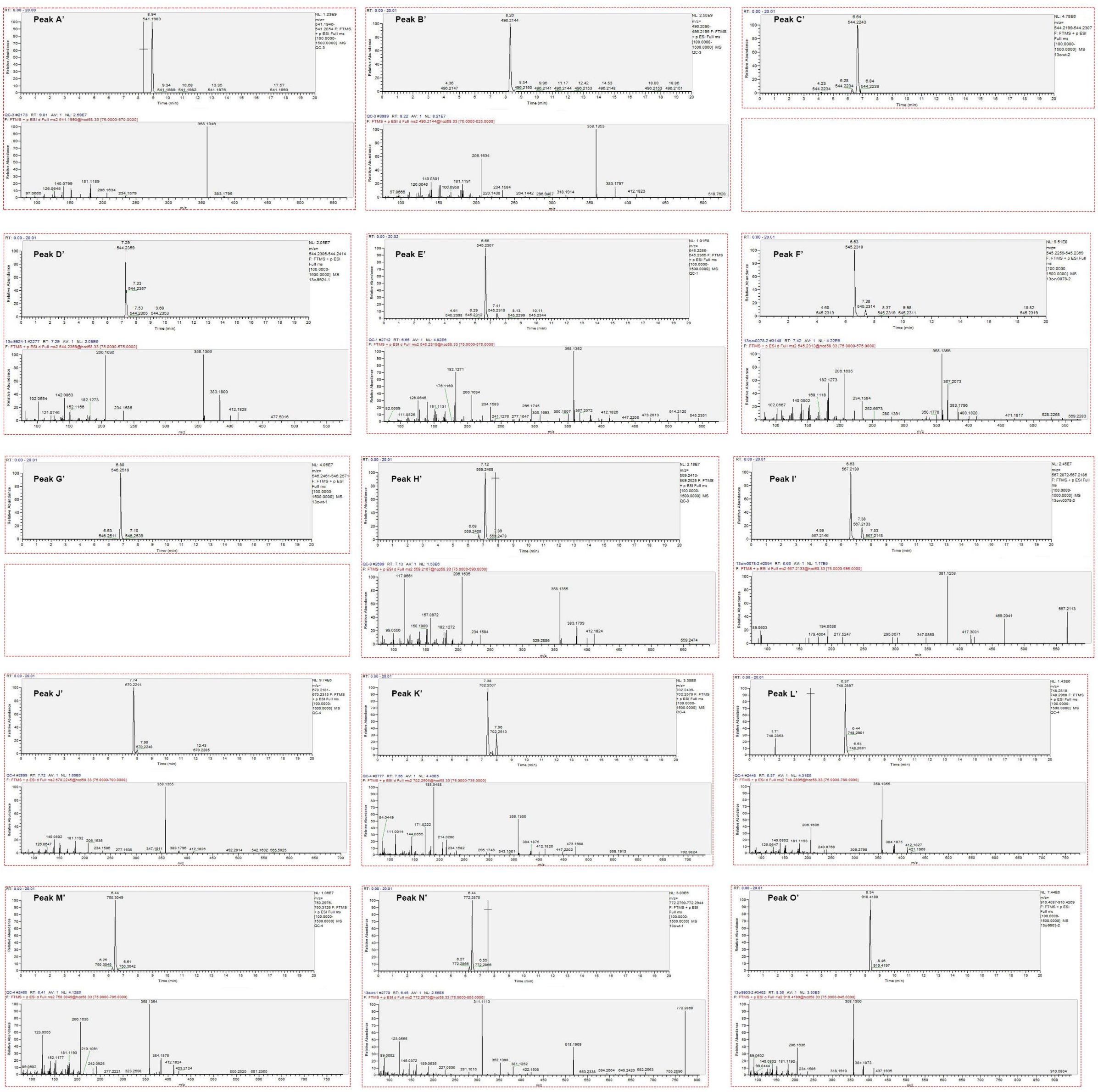


**Fig S5. LC peak profiles of the DLM metabolites shown in Figure 3a, and the corresponding ion spectra from DLM (A)- and $^{13}C_6$-DLM (B)-treated Mtb.** Product ion spectra of peak C' and peak G' were not detected in any samples.

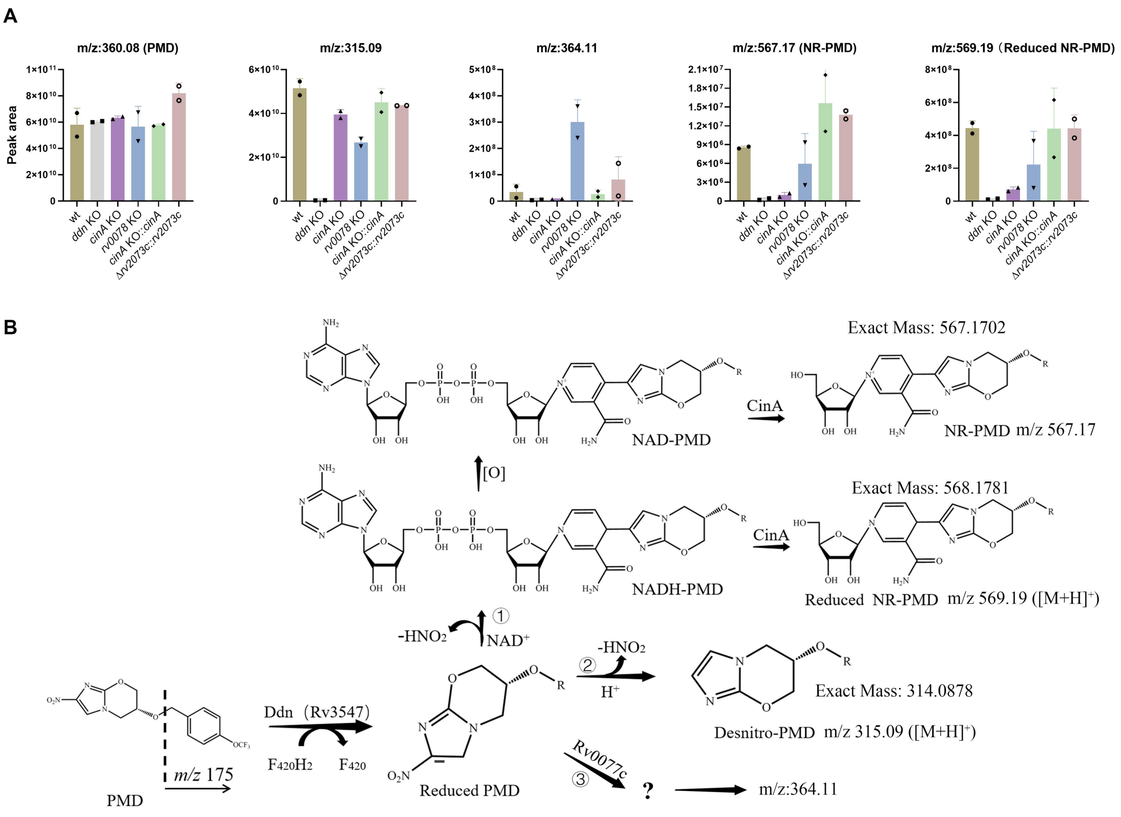


**Fig S6. Identification of specific metabolites of PMD. (A)** Quantification of the relative abundance of the corresponding metabolites from PMD-treated Mtb mutants. Data are expressed as the mean ± SD of two cultures. **(B)** Proposed mechanism of PMD metabolism in Mtb. The structures of NR-PMD, reduced NR-PMD and desnitro-PMD were proposed based on detected precursor ion m/z and the relative DLM metabolites.

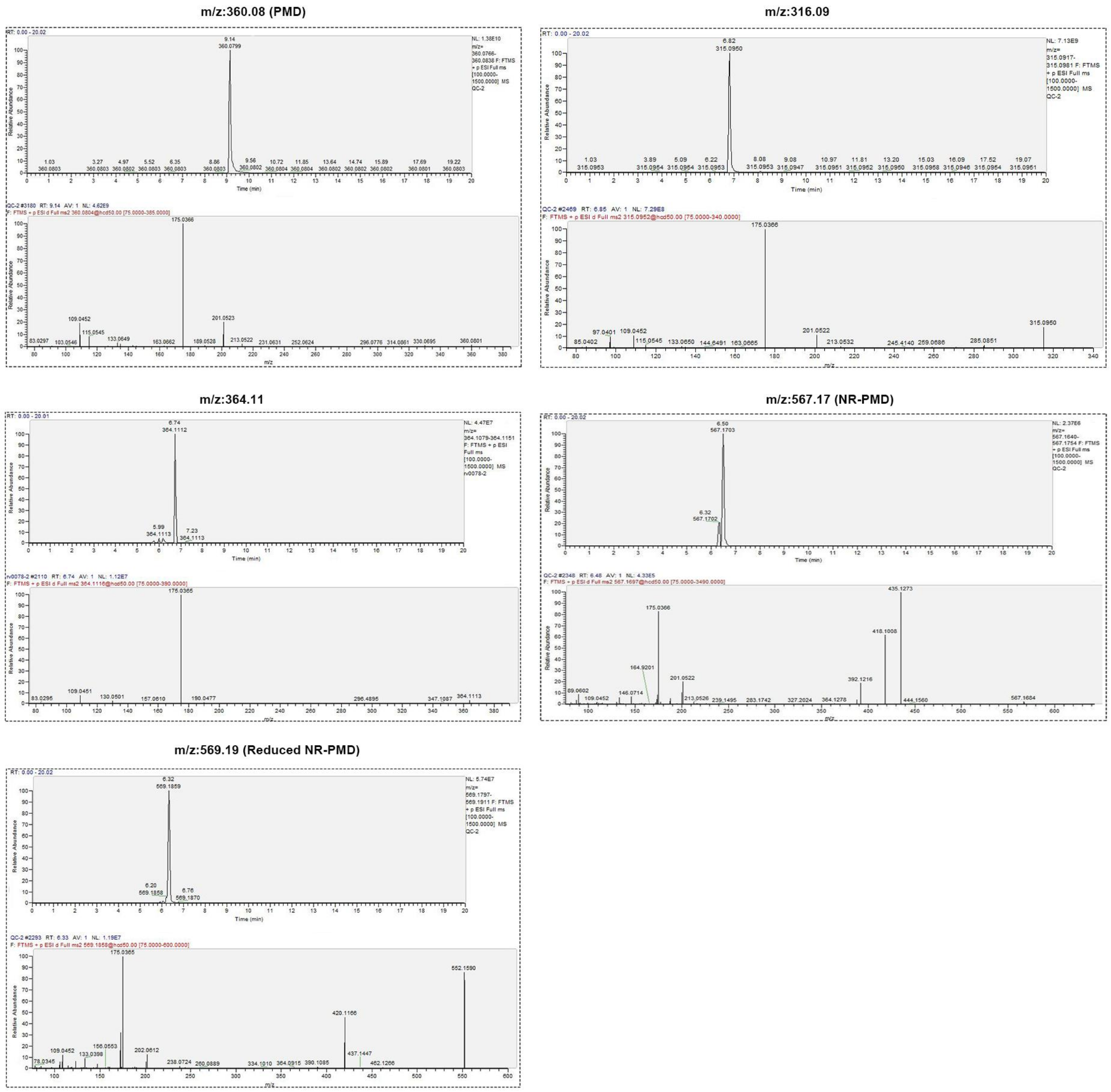


**Fig S7. LC peak profiles of the PMD metabolites shown in Figure S6A, and the corresponding ion spectra from PMD-treated Mtb.**

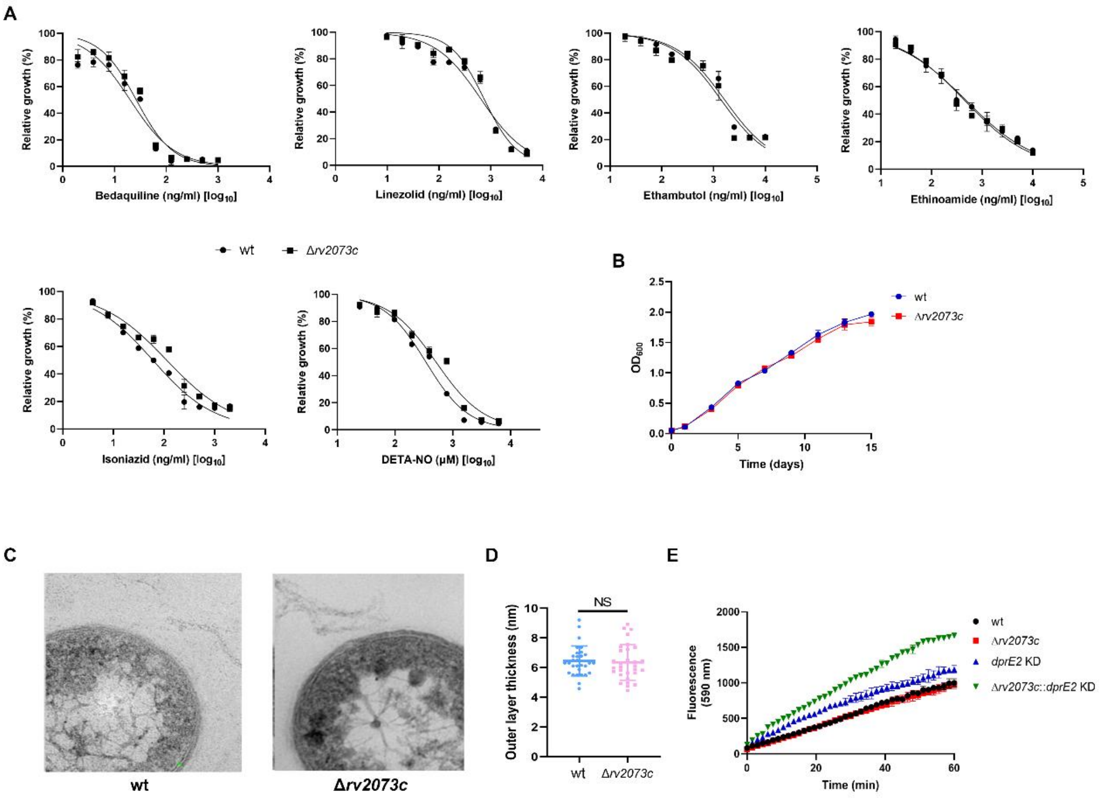


**Fig S8. Rv2073c is an intrinsic resistance gene specific to DLM and PMD. (A)** Mutation of *rv2073c* did not alter susceptibility of *Mtb* to other antibiotics. Dose-response curves of the indicated strains to other known antibiotics. Data are expressed as the mean ± SD of triplicate samples, and are representative of at least two independent experiments. **(B)** Growth curves of the indicated strains. **(C)** Transmission electron microscopy (TEM) images of *rv2073c* mutants and wild-type Mtb show no difference in the thickness of the outer cell envelope. **(D)** Comparison of cell-wall outer layer thickness (as measured by TEM) of *rv2073c* mutants and wild-type Mtb. Each dot represents the thickness of one Mtb cell, and error bars show the standard deviation. **(E)** Uptake of EtBr by wild-type, Δ*rv2073c*, *dprE2* CRISPRi knockdown strains and *dprE2* CRISPRi knockdown in *rv2073c* mutant strains. Data are expressed as the mean ± SD of triplicate samples, and are representative of at least two independent experiments.

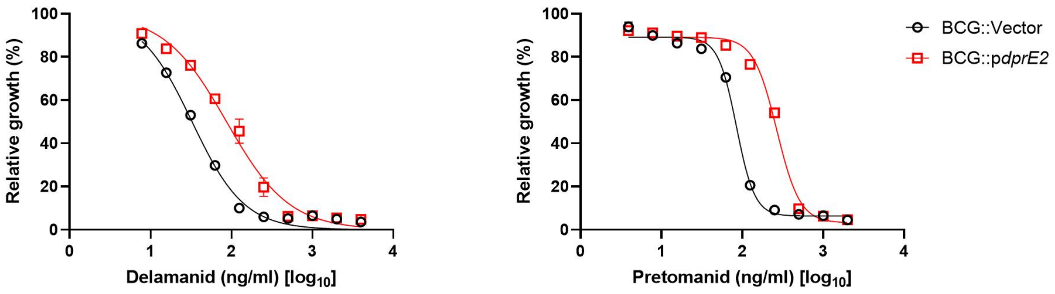


**Fig S9. Susceptibility of the indicated BCG strains to DLM and PMD.** The $MIC_{50}$ was defined as the concentration of compound that inhibited bacterial growth by 50%, and was analyzed by a nonlinear fit model in GraphPad Prism 9.0. Data are expressed as the mean ± SD of triplicate samples, and are representative of at least two independent experiments.

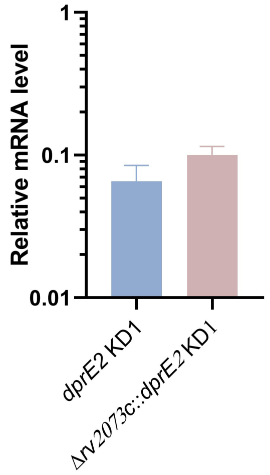


**Fig S10. Quantification of *dprE*2 mRNA levels by RT-qPCR.** Mtb H37Ra wild type and *rv2073c* deletion mutant harboring *dprE2* CRISPRi plasmid or control vector were grown with ATc for ~3 days before RNA collection. The relative mRNA level was determined by comparing the mRNA levels of *dprE2* in strains with CRISPRi knockdown to those in strains with control vector. qPCR data were collected from three independent experiments with three technical replicates per experiment. Error bars show the standard deviation from three independent experiments.

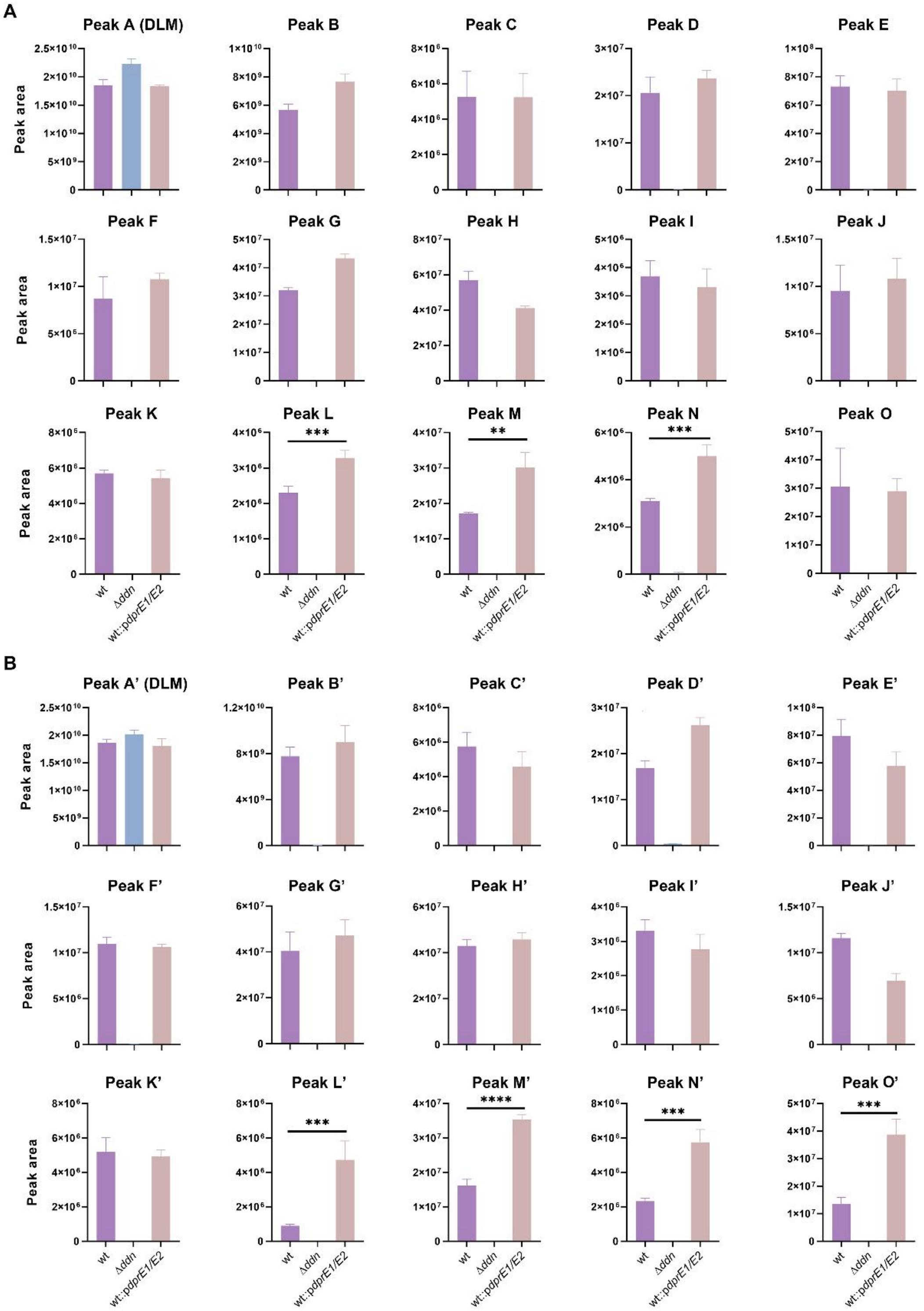


**Fig S11. Quantification of the relative abundance of the corresponding metabolites from DLM-treated (A) and $^{13}C_6$-DLM-treated (B) Mtb strains**. Data are expressed as the mean ± SD of three cultures. Statistical significance was assessed by one-way ANOVA. ** $P < 0.01$; *** $P < 0.001$; **** $P < 0.0001$.

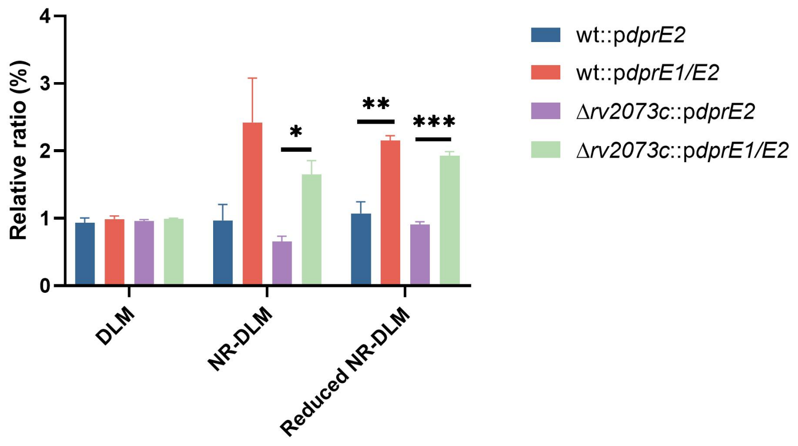


**Fig S12. The Relative Abundance Ratio of Corresponding Metabolites in DLM-treated Indicated Strains.** The relative ratio was determined by comparing the metabolites in over-expressing strains to those in their corresponding background strains. Data are expressed as the mean ± SD of three cultures. Statistical significance was assessed by two-way ANOVA with Dunnett's multiple comparison test. * $P < 0.05$; ** $P < 0.01$; *** $P < 0.001$.

**Supplementary Tables**

**Table S1:** Genetic Hits from the Genome-wide CRISPR-KO Screen in DLM, PMD or DETA/NO treatment.

**Table S2:** Genetic Hits from the Genome-wide CRISPRi Screen in DLM, PMD or DETA/NO treatment.

**Table S3:** Lipidomics analysis of the *rv2073c*-deleted and wild-type *M. tuberculosis* H37Ra strains.

**Table S4:** Metabolomics analysis of the *rv2073c*-deleted and wild-type *M. tuberculosis* H37Ra strains.

**Table S5**: Lipidomics analysis of the Δ*rv2073c*::*dprE2* KD and *dprE*2 KD of *M. tuberculosis* H37Ra strains.

**Table S6.** List of identified nonsynonymous mutations in *rv0078* from clinical isolate WGS database.

**Table S7:** List of sgRNAs and primers used in this work.

### sgRNAs used in this work

| sgRNA ID | sgRNA targeting sequence (5'–3') | Gene targeted |
|---|---|---|
| Non-Targeting control | AAGCTGTTATCCACAGCGACC | NA |
| rv2073c sgRNA | GTTGATCGGCGTTCCGCGCGG | *rv2073c* |
| rv0078 sgRNA | GCCCAGCACGACGGGCGCATCCA | *rv0078* |
| ddn sgRNA | ATGACCCGCCCACCGTCGCG | *ddn* |
| cinA sgRNA | GCTGGTGCTGGACGACGAGCT | *cinA* |
| rv0077c sgRNA | GTCCTTGAGCTCGAGCGCGG | *rv0077c* |
| rv0077c/rv0078 pair sgRNAs | ACTGGTCGGCGAGCCGGTCC | *rv0077c* |
| | GACGACCCCAAGCGCGCCCG | *rv0078* |
| dprE2 sgRNA | GATCTCGGAGGTGCCACCGA | *dprE2* |
| embA sgRNA | AGGTCGCGGTGGTTTGGTTCA | *embA* |

### Primers used in this work

| Name | Sequence (5'–3') |
|---|---|
| primer F for sgRNA seq | CTCTGACCAGGGAAAATAGCCC |
| primer R for sgRNA seq | GCCATTGATAATGCTCTTCATCCC |
| primer F for KO cycle1 sgRNA seq | AAGATCGACGAGTTGGCCTT |
| primer R for KO cycle1 sgRNA seq | TTTCTGCCGAACAAACGCAT |
| primer F for rv2073c | CGGGATCCAGTGGACGACACGGGCGCT |
| primer R for rv2073c | CCCAAGCTTTCATCGCGGCATCCTGCGC |
| primer F for rv0077c | CGGGATCCAATGTCGACGATCGACATTAG |
| primer R for rv0077c | CCCAAGCTTACAGCGGGCATTCTAAGTCC |
| primer F for cinA | CGGGATCCAATGGCGGTGAGCGCACGTGC |
| primer R for cinA | CCCAAGCTTCTAGGGTGAGCCCGGGATAC |
| primer F for dprE2 | CGGGATCCAATGGTTCTTGATGCCGTAGG |
| primer R for dprE2 | CCCAAGCTTTCAGATGGGCAGCTTGCGGAAG |
| primer F for dprE1/E2 | TGCGTTTAATACTGTTTCGATGATCCGATCGGAGTCT |
| primer R for dprE1/E2 | TTAATTAGCTAAAGCTTTCAGATGGGCAGCTTGCGGA |

### qPCR primers used in this work

| Gene name | Forward primer (5'–3') | Reverse Primer (5'–3') |
|---|---|---|
| *dprE2* | ATTGACCGTCGACAAGGAGT | TGACGCAACACCATCATGAC |
| *sigA* | GGTGATTTCGTCTGGGATGAA | GCTACCTTGCCGATCTGTTTG |

**Table S8:** Strains constructed in this work.

| Strain | Gene name | Deletion size | Deletion position | Annotation[c] |
|---|---|---|---|---|
| H37Ra[a] | *rv2073c* | 350 bp | 2340088-2340437 | Δ*rv2073c* |
| | *ddn* | 11 bp | 3995715-3995725 | *ddn* KO |
| | *rv0078* | 10 bp | 88232-88241 | *rv0078* KO |
| | *rv0077c* | 181 bp | 87561-87741 | Δ*rv0077c* |
| | *cinA* | 16 bp | 2157877-2157892 | *cinA* KO |
| | *rv0077c/0078* | 1408 bp | 87027-88434 | Δ*rv0077c/0078* |
| H37Rv[b] | *rv2073c* | 444 bp | 2330414-2330857 | Δ*rv2073c* |
| | *rv0078* | 16 bp | 86874-86889 | *rv0078* KO |

[a] The reference genome of H37Ra strain is NC_009525.1.

[b] The reference genome of H37Rv strain is NC_000962.

[c] Gene deletions of large size are denoted by the symbol Δ, whereas minor deletions are represented by the abbreviation KO.
